# Temporal genomic contrasts reveal rapid evolutionary responses in an alpine mammal during recent climate change

**DOI:** 10.1371/journal.pgen.1008119

**Published:** 2019-05-03

**Authors:** Ke Bi, Tyler Linderoth, Sonal Singhal, Dan Vanderpool, James L. Patton, Rasmus Nielsen, Craig Moritz, Jeffrey M. Good

**Affiliations:** 1 Museum of Vertebrate Zoology, University of California, Berkeley, California, United States of America; 2 Computational Genomics Resource Laboratory (CGRL), California Institute for Quantitative Biosciences (QB3), University of California, Berkeley, California, United States of America; 3 Department of Integrative Biology, University of California, Berkeley, California, United States of America; 4 Division of Biological Sciences, University of Montana, Missoula, Montana, United States of America; 5 Research School of Biology and Centre for Biodiversity Analysis, Australian National University, Canberra, ACT, Australia; 6 Wildlife Biology Program, University of Montana, Missoula, MT, United States of America; University of Wyoming, UNITED STATES

## Abstract

Many species have experienced dramatic changes in their abundance and distribution during recent climate change, but it is often unclear whether such ecological responses are accompanied by evolutionary change. We used targeted exon sequencing of 294 museum specimens (160 historic, 134 modern) to generate independent temporal genomic contrasts spanning a century of climate change (1911–2012) for two co-distributed chipmunk species: an endemic alpine specialist (*Tamias alpinus*) undergoing severe range contraction and a stable mid-elevation species (*T*. *speciosus*). Using a novel analytical approach, we reconstructed the demographic histories of these populations and tested for evidence of recent positive directional selection. Only the retracting species showed substantial population genetic fragmentation through time and this was coupled with positive selection and substantial shifts in allele frequencies at a gene, *Alox15*, involved in regulation of inflammation and response to hypoxia. However, these rapid population and gene-level responses were not detected in an analogous temporal contrast from another area where *T*. *alpinus* has also undergone severe range contraction. Collectively, these results highlight that evolutionary responses may be variable and context dependent across populations, even when they show seemingly synchronous ecological shifts. Our results demonstrate that temporal genomic contrasts can be used to detect very recent evolutionary responses within and among contemporary populations, even in the face of complex demographic changes. Given the wealth of specimens archived in natural history museums, comparative analyses of temporal population genomic data have the potential to improve our understanding of recent and ongoing evolutionary responses to rapidly changing environments.

## Author summary

Museum specimens represent an irreplaceable archive that can be used to understand how species respond to rapid environmental change. We recovered genomic data from archived samples spanning a century of climate change in co-distributed declining versus stable species of montane chipmunks. Applying novel statistical methods, we find evidence for strong positive selection on a physiologically relevant gene despite increased population fragmentation in the declining species. Our results reveal rapid evolutionary responses, but also highlight that genetic adaptation has been insufficient to prevent range collapse in this endemic alpine species. These findings illustrate how biological archives can be used to pinpoint genetic responses through time to better understand how species are responding to rapidly changing environments.

## Introduction

Rapid environmental change threatens global biodiversity and has led to population declines in many species [1–5]. Although phenotypic plasticity may enable populations to track rapidly changing climates, evolutionary adaptation will often be essential for long-term persistence [6]. Disentangling plasticity from evolutionary responses ultimately requires resolving the genetic basis of adaptation. However, it remains challenging to differentiate recent or ongoing positive selection from stochastic genetic drift in contemporary populations that are also undergoing extreme demographic changes [7, 8]. Natural history museum collections may hold the key to overcoming many of these difficulties by providing crucial temporal information on species distributions, phenotypes, and population genetic variation spanning periods of recent environmental change [9–11]. Temporal genomic contrasts have begun to yield powerful insights into recent evolutionary responses in humans [12, 13] and other species [14–17], indicating that genetic analyses of biological archives will be an effective tool for understanding evolutionary responses to rapid anthropogenic climate change [18].

Using contrasts between early 20^th^ century and modern museum surveys, Moritz and colleagues [1] showed that the ranges of several high elevation small mammal species in the Yosemite National Park (YNP) region of the Sierra Nevada mountains (California, USA) have retracted upward over the past century. This and associated studies [19, 20] demonstrated the potential of using museum archives to understand species and community-level ecological responses during periods of recent climate change. Contemporary range shifts towards higher latitudes, elevations, or both have now been documented in many terrestrial species [5, 19, 21, 22], and are generally thought to reflect direct and indirect population responses to warming temperatures [22, 23]. However, these works have also highlighted that closely related species can differ markedly in the magnitude and direction of their ecological responses, for reasons that are often not clear [1, 20]. Currently, we know very little about how recent range shifts have affected evolutionary processes within species, or the extent to which evolutionary genetic responses have been synchronous within and between co-distributed species.

**Fig 1:**
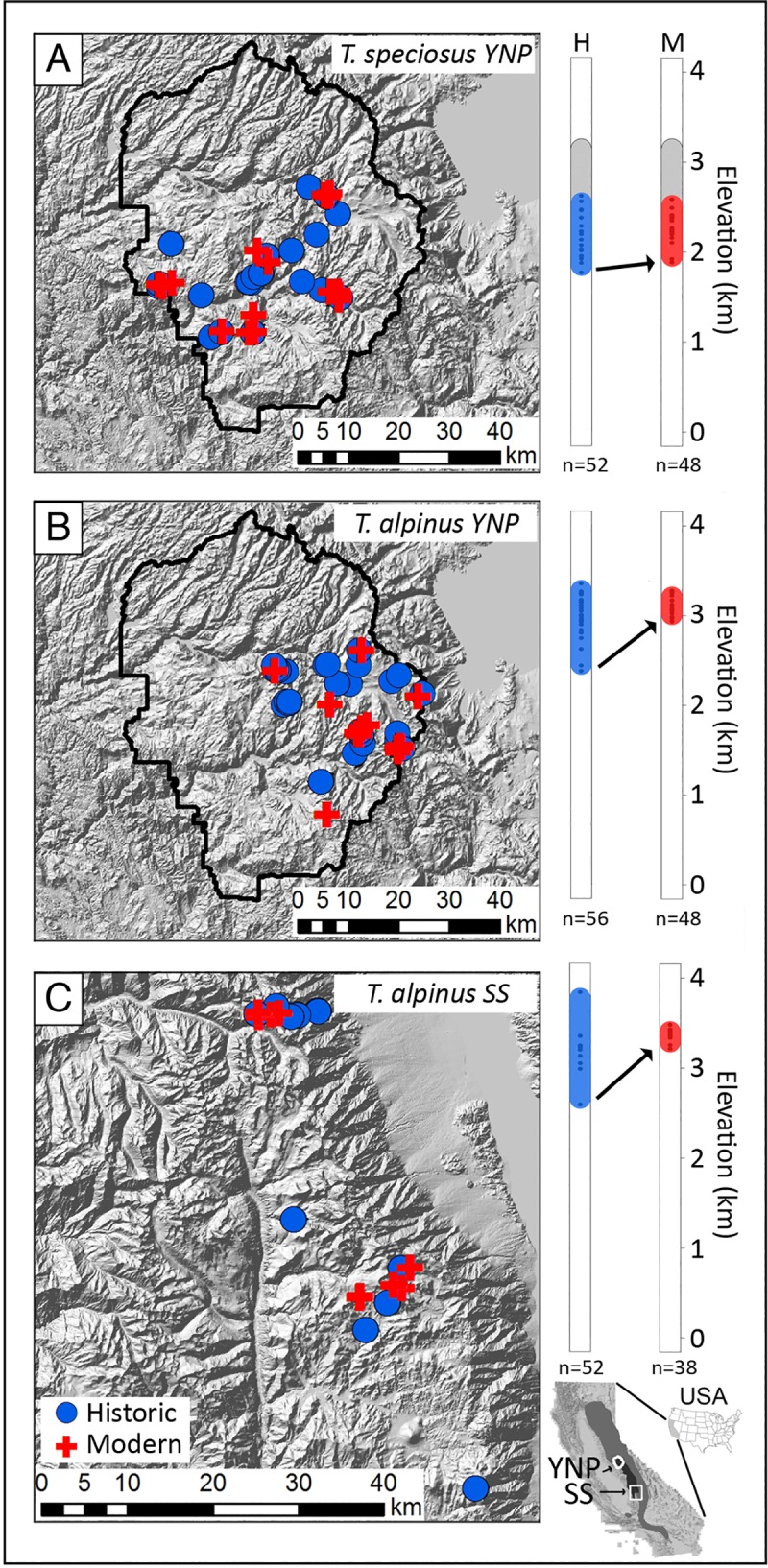
Historic and modern sampling localities. *Tamias speciosus* and *T*. *alpinus* specimens were collected from Yosemite National Park **(A, B)** and the Southern Sierras **(C)**. Historical sampling localities (1911–1916) are shown in filled blue circles and modern (2003–2012) in filled red crosses. The vertical bar plots in the right panel show the elevation (kilometers) range of the species over the past century with sequenced samples indicated by colored dots within the bars. Upper elevation limits were determined for *T*. *speciosus* (shown in gray), but sequenced samples were selected from mid-elevation sites where sufficient historical and modern samples were available. The trapping resurvey was not designed to ascertain upper limits for *T*. *alpinus* from either transect area. Sequencing sample sizes (n) are indicated under the elevation bars. While *T*. *speciosus* maintained a stable range, *T*. *alpinus* populations have severely contracted upwards in elevation (indicated with arrows) throughout their distribution [20]. The inset (bottom right) USA map shows the ranges of *T*. *alpinus* (dark gray) and *T*. *speciosus* (gray) with the state of California with Yosemite National Park (YNP, northern) and the Southern Sierras (SS, southern) study areas are indicated with arrows and outlined in white.

Here we focus on two chipmunk species within the YNP montane mammal community that show different ecological and phenotypic responses over the last century of climate change (Fig 1). Western chipmunks have long been considered models for niche partitioning by elevation and habitat type [24–26]. The alpine chipmunk (*Tamias alpinus*) is an ecological specialist endemic to the high elevation alpine habitats of the Sierra Nevada Mountains. The lodgepole chipmunk (*T*. *speciosus*) occurs more broadly across mid- to high-elevation subalpine coniferous forests of California. *Tamias alpinus* has undergone severe range contraction driven by extirpation of lower elevation populations [1, 20] combined with pronounced shifts in diet and cranial morphology [27] across the alpine zone of YNP and elsewhere in the Sierra Nevada mountains. Spatial modeling of current versus historical ranges across YNP suggests that the strong upward contraction of *T*. *alpinus* is best explained by increases in minimum winter temperatures; competing models including co-occurrence with other species of chipmunks or changes in the distribution of preferred vegetation types did not improve prediction of the observed range contraction in this species [28, 29]. That increasing minimum temperature alone had the strongest impact makes sense in that there has been little change in vegetation across the high elevation talus slopes preferred by this species. By contrast, range contraction in a mid-elevation chipmunk species, *T*. *senex*, is best explained by changes in its preferred vegetation types [28, 29]. For YNP *T*. *alpinus*, there is also evidence of strong directional selection on skull morphology over the past century [30], whereas the range, diet, and morphology of the partially overlapping lodgepole chipmunk (*T*. *speciosus*) has remained relatively stable within YNP [20, 27, 31]. As is generally the case [32], it remains unclear why the montane specialist, *T*. *alpinus*, has contracted during the past century whereas its more widely-distributed congener, *T*. *speciosus*, has not.

These two species set up a natural contrast, allowing us to understand how differing ecological responses during periods of rapid environmental change correspond to differing evolutionary responses. A previous temporal survey of eight microsatellite markers revealed increased subdivision and declining allelic diversity in YNP *T*. *alpinus*, but no significant changes in overall population genetic variation of *T*. *speciosus* over the same interval [33]. Synthesizing descriptions of phenotypic [30], behavioral [31], and genetic variation [33] into a detailed understanding of demographic and evolutionary responses in these species requires genomic data. Towards this end, Bi et al. [9] used a custom exon capture platform to enrich and sequence ~11,000 exons (~4 Megabases or Mb across 6,249 protein-coding genes) from 20 early 20^th^ century and 20 modern YNP *T*. *alpinus*. These genome-wide data confirmed signatures of increasing population subdivision in this retracting species and illustrated the potential for targeted genomic experiments to generate high quality data from archived specimens. However, neither of these preceding analyses had sufficient sampling to determine whether evident increases in population structure were due to reductions in local population size, migration rates, or both.

Here, we build on these previous works [9, 33] by generating ~9.4 Mb of targeted exome sequence data from 303 chipmunks (194 *T*. *alpinus*, 100 *T*. *speciosus*, and 9 samples from 4 other species). We used these comparative population genomic data to quantify evolutionary responses over the past century at two scales (Fig 1). First, we sequenced 96 modern (48 *T*. *alpinus* and 48 *T*. *speciosus* collected between 2003–2008) and 108 historic (56 *T*. *alpinus*, 52 *T*. *speciosus* collected in 1915–1916) samples collected across geographic transects in YNP for the focal species. Second, we generated an independent geographic transect of 38 modern (2003–2012) and 52 historic (1911–1916) *T*. *alpinus* in the Southern Sierras (SS), where this species also shows range contraction [20], to test to what extent evolutionary responses across the range of this declining alpine specialist have been consistent. To analyze these temporal data, we developed a novel analytical framework based on Approximate Bayesian Computation (ABC) that allowed us to quantify changes in population sizes and migration rates in the context of demographic history, and then to localize positive selection on standing genetic variation at specific genes. Our results provide new insights into recent evolutionary responses in this system and demonstrate how a genomic time-series approach can be broadly applied to other archived specimens to improve understanding of evolutionary responses over the past few centuries.

## Results and discussion

### Targeted enrichment of genomic data spanning a century of climate change

High sequencing coverage is necessary to reliably genotype ancient DNA samples [34] due to extensive DNA degradation [35]. This persistent technical challenge makes whole genome resequencing of historic mammalian populations impractical, especially in species without high-quality reference genomes (e.g., *Tamias*). Therefore, we designed a custom targeted capture to enrich and sequence exons from over 10,107 protein-coding genes (9.4 Megabases or Mb total) in 294 *T*. *alpinus* and *T*. *speciosus* samples (S1 Table) and nine samples from four other chipmunk species (total n = 303). We sampled geographic transects of modern and historic (~100 year-old) populations in YNP for both species as well as an independent SS transect of *T*. *alpinus*, where this species has also contracted [20]. An average of 49 individuals were sequenced per population (Fig 1). This design allowed us to (i) compare stable and retracting species within the same montane mammal community (YNP), and to (ii) determine to what extent the same evolutionary responses have occurred across two transects (YNP and SS) spanning the latitudinal range of the range-retracted species *T*. *alpinus*.

Exome enrichment was highly specific (90–93% of cleaned reads on target) and sensitive (>92% of the target regions sequenced), resulting in high coverage of targeted regions (26–35× average individual coverage per population, S2 Table). Although historic DNA samples are notorious for poor technical performance, all targeted *T*. *speciosus* and *T*. *alpinus* individuals yielded moderate to high coverage data with similar capture success between modern and historic samples. Analysis of mitochondrial DNA indicated that empirical error rates were ~fourfold higher in historic (0.16%) versus modern samples (0.04%), due primarily to DNA damage typical of century-old museum samples [9, 36]. Although nucleotide misincorporations associated with deamination of methylated cytosine bases (C-to-T and G-to-A) were most common near the ends of DNA fragments, such changes remained elevated throughout the sequence (S1 Fig). Therefore, we applied several quality filters to remove all putative misincorporations and to mitigate other common sources of genotyping error [9] (S3 Table). All filters were uniformly applied to historic, modern, and simulated data (see below) to facilitate comparisons across time periods and species. After filtering, we identified 20,395, 10,395, and 10,954 high-quality single nucleotide polymorphisms (SNPs) in YNP *T*. *speciosus*, YNP *T*. *alpinus*, and SS *T*. *alpinus*, respectively.

### Population genetics and demographic inference

Upwards range contraction in montane environments can lead to increased population structure and reduced genetic diversity due to decreased population size. We observed a consistent trend towards relatively minor reductions in nucleotide diversity in modern versus historic samples of *T*. *alpinus* and *T*. *speciosus* estimated at two spatial scales: metapopulations (e.g., YNP or SS) and demes of spatially clustered sampling localities (θ_π_ and θ_W_; S2 Fig, S4 Table), consistent with prior results [33]. We then quantified the degree of population genetic structure within each species by estimating the global fixation index (F_ST_) for historic and modern populations. Within YNP, population structure was relatively low overall but increased nearly two-fold in modern *T*. *alpinus* (F_ST,historic_ = 0.032, F_ST,modern_ = 0.058), consistent with increased population fragmentation as this species has contracted upwards [33]. By contrast, population structure in *T*. *speciosus* increased only slightly over time (F_ST,historic_ = 0.027, F_ST,modern_ = 0.030). We detected very little uncertainty in our estimates of the site frequency spectrum (SFS), and estimates of global F_ST_ showed non-overlapping 0.95 bootstrapped confidences intervals for all pairwise temporal contrasts (S4 Table). Collectively, these patterns suggest that the stronger increase in F_ST_ observed for YNP *T*. *alpinus* is not simply explained by reductions in per deme nucleotide diversity, which are of similar magnitude across YNP and SS *T*. *alpinus* and YNP *T*. *speciosus* (S2 Fig).

**Fig 2:**
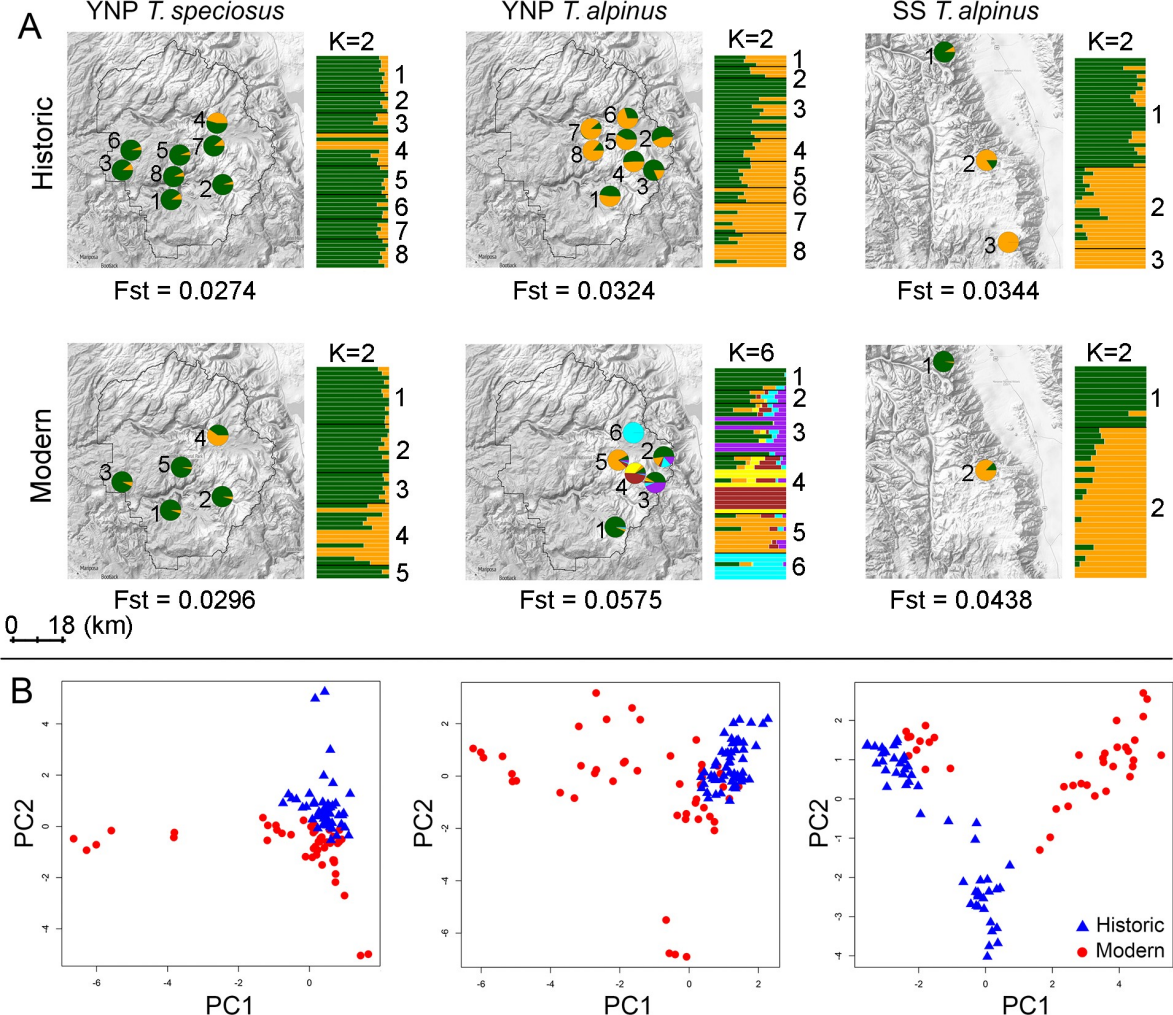
Temporal and spatial population genetic structure. **(A)** Genetic clustering by general sampling locality (left, pie charts) and individual (bar graph to the right of each map) across historic and modern samples based on ngsAdmix analyses. Each individual is partitioned into colored segments that indicate cluster membership. Pie charts represent the sum of all individuals’ membership in each cluster at each general locality on the map. Proximate individual sample localities were pooled for clarity following Rubidge and colleagues [33]. The inferred best number of clusters (K) is shown on the top of each bar graph. Global F_ST_ estimates between localities are indicated at the bottom of each map. **(B)** Each data point in the PCA plot represents an individual specimen. The first and second two principal components (PCs) explain 3.7% and 2.6% of the total genetic variance.

To further evaluate changes in the spatial patterning of population genetic structure, we used NGSadmix to conduct a maximum likelihood (ML) analysis of historic and modern population structures [37]. We detected a substantial increase from two to six genetic clusters for YNP *T*. *alpinus*, compared to stable overall population structure (K = 2 across both time points) within *T*. *speciosus* (Fig 2A; S5 Table). Principal component analyses of these data also indicate less genetic similarity among modern YNP *T*. *alpinus* samples (Fig 2B). These genome-wide estimates incorporate genotype uncertainty to provide an accurate overview of changes in genetic diversity and structure over recent timescales. Although consistent in the direction of change, the increases in YNP *T*. *alpinus* population genetic structure appear even more striking than previously detected using lower resolution data [33, 38]. By contrast, there was minimal structure in *T*. *speciosus* within the same ecosystem whether considering a few microsatellite loci [33] or thousands of SNPs (Fig 2A).

Consistent with patterns of increased population genetic structure, spatial models of occurrence indicate that range contraction has reduced local connectivity between suitable habitat patches for YNP *T*. *alpinus* [33]. Given that range contraction has also been detected at the southern limit of the range of *T*. *alpinus* [20], we next tested to what extent similar temporal signatures of increased genetic structure were apparent in SS populations (Fig 1). Population structure in the SS transect has also increased (F_ST,historic_ = 0.034, F_ST,modern_ = 0.044), but to a lesser extent than observed in YNP, and with no overall increase in the number of distinct genetic groups (modern and historic K = 2; Fig 2A). This suggests some variation in the local genetic consequences of seemingly synchronous, range-wide contractions. However, we note that our power to detect changes in overall population structure may have been limited by the fact that historic and modern sampling localities were more spatially clustered in SS when compared to YNP (Fig 1).

Rapid range shifts could also increase the likelihood of hybridization if there are changes in the degree of sympatry or in relative densities among closely related species [39, 40]. Occasional hybridization appears to be common in western chipmunks [41], including evidence for ancient mitochondrial introgression from the broadly distributed least chipmunk, *T*. *minimus*, into *T*. *speciosus* [42]. The alpine chipmunk is very closely related to *T*. *minimus* with evidence of historical gene flow [38], raising the possibility that recent range fluctuations have induced hybridization between *T*. *alpinus* and either *T*. *minimus* or *T*. *speciosus*. However, we found no evidence for recent appreciable nuclear gene flow between *T*. *alpinus* and adjacent populations of *T*. *speciosus* or with neighboring (lower elevation) populations of *T*. *minimus* (S3 Fig). Thus, recent range collapse does not appear to have led to the breakdown of reproductive barriers between this high elevation endemic and other co-distributed species [38]. We did, however, detect nuclear introgression from *T*. *speciosus* into at least one *T*. *minimus* sample (S3 Fig), suggesting that reproductive isolation remains incomplete between these species despite strong ecological partitioning [26].

These basic descriptions of genetic variation expand the scope and resolution of previous analyses of a few microsatellite loci genotyped in *T*. *alpinus*, *T*. *minimus*, and *T*. *speciosus* [33, 38], and from more limited exon capture data from *T*. *alpinus* [9]. However, disentangling changes in migration versus local effective population size, and identifying genes under positive selection in the context of recent demographic change, requires more comprehensive analyses. Therefore, we developed a novel analytical framework to fully exploit our temporal dataset. Traditional population genetic analyses often assume even sampling across space and time, yet studies using museum specimens are typically imbalanced because of limited availability of samples. ABC is well suited for demographic inference under such circumstances because biased temporal sampling and sample processing can be simulated for populations that have experienced complex demographic histories. Accordingly, simulations can be filtered in the same way as observed data (e.g., removal of SNPs associated with errors in historic DNA), permitting meaningful comparisons between expected and observed results. A major difficulty of ABC is the choice of statistics that sufficiently describe demographic parameters of interest. Multiple, jointly informative, summary statistics are often used to sufficiently estimate parameters while reducing the risk of any particular statistic biasing the results [43]. The site frequency spectrum (SFS) is often an optimal choice for fitting demographic histories since many commonly used summary statistics can be derived from it. In practice, high dimensionality and low count categories of joint site frequency spectra make them difficult to fit. Consequently, we developed an effective means of fitting binned two-dimensional SFS (2D-SFS) using an ABC framework designed to infer population histories from serially sampled metapopulations (S4 Fig).

**Fig 3:**
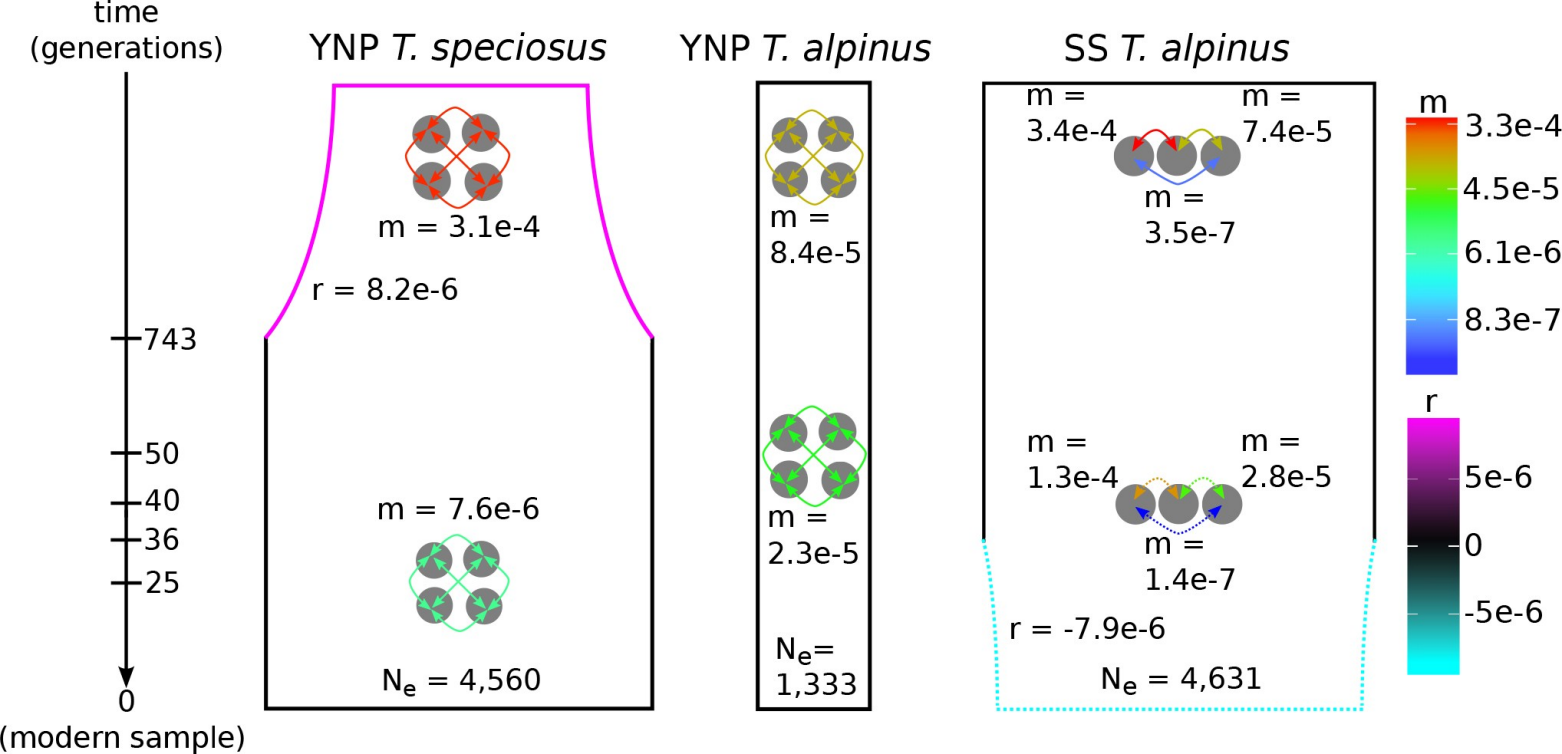
Population histories inferred with Approximate Bayesian Computation. Population histories fitted with ABC showing the general topology and posterior median demographic parameter values averaged across the best fitting models for YNP *T*. *speciosus*, YNP *T*. *alpinus*, and SS *T*. *alpinus*, respectively. The fitted parameters are the modern effective number of individuals per deme (Ne), migration rates (m), intrinsic growth rates (r), and the timing of demographic events (1 generation = 1 year). History widths are proportional to the deme effective sizes though time. The four-deme depiction (YNP *T*. *alpinus* and *T*. *speciosus*) represents histories fitted with an island model having equal pairwise migration rates between all demes, and where the actual number of islands equaled the number of sampled demes. Three SS *T*. *alpinus* demes were sampled and modeled as islands with migration rates being allowed to vary between different pairs of demes. The events with dashed lines for SS *T*. *alpinus* are relatively uncertain (see S1 Text), but if any demographic changes did occur they are as shown (note that a bottleneck is likely to have been weak and the size is not to scale).

We constructed 2D-SFS for each of the three pairwise temporal contrasts by pooling individuals across sampling localities within YNP or SS into a single metapopulation per time period (S5 Fig). While allele frequencies should be highly correlated over such short time scales, the overall shape of the joint spectrum should change in predictable ways in response to various population-level processes. We fitted multiple demographic models to the 2D-SFS (S6 Fig; S7 Fig) describing population size (constant size, bottlenecks, expansions) and connectivity (migration) among subpopulations or demes representing spatially clustered localities (see S6–S9 Tables and S1 Text for details on model selection, evaluation, and inference). The best fitting population history for YNP *T*. *alpinus* was characterized by relatively small but constant deme effective sizes through time (~1,350 individuals) and an approximately threefold decrease in migration within the past ~90 years (Fig 3). This fitted demographic model fits well with our observation of increased population structure in YNP *T*. *alpinus* (Fig 2) despite only minor reductions in nucleotide diversity per deme through time (S2 Fig, S4 Table). In contrast, both SS *T*. *alpinus* and YNP *T*. *speciosus* were found to have much larger effective deme sizes (~4,600 and ~4,560 individuals respectively) and higher migration rates overall. Consistent with the observed increase in F_ST_, SS *T*. *alpinus* showed some evidence for a recent, very small decline in effective size (Fig 3). YNP *T*. *speciosus* was the only population that showed a clear signature of size change, albeit related to a historic (pre 20^th^ C) population expansion (Fig 3).

We found that modern samples tended overall to be less genetically similar to each other than did historic samples in all three comparisons (Fig 2B; S8 Fig). Consistent with this observation, the fitted demographic histories for each species and transect support a recent (< 90 years ago) decrease in migration among demes (Fig 3; see S1 Text, S6 Table). Decreased migration is expected if climate change is broadly affecting the amount of connectivity between suitable habitat patches available to species within this montane community [33]. However, as noted above, substantially increased genetic structuring was most evident in YNP *T*. *alpinus* (Fig 2A). Long-term changes in gene flow should ultimately affect the genetic composition of metapopulations across these landscapes. It is possible that higher overall migration rates and larger effective population sizes have so far buffered the population genetic effects in *T*. *speciosus* and SS *T*. *alpinus*. However, genetic structure could accumulate over time according to the inferred histories.

These results emphasize the utility of high-resolution demographic inference from genomic data not only for reconstructing population histories, but also as a potentially powerful conservation management tool [18, 44, 45]. An ABC framework is generalizable to other temporally sampled genetic datasets, allowing high-resolution inference into demographic histories over shallow evolutionary timescales that are relevant to recent anthropogenic climate change. In species of conservation concern, signatures of reduced migration could be used to motivate introductions between populations prior to significant genetic erosion, buffering against the future loss of genetic diversity and the accumulation of deleterious variation [46]. The benefits of such proactive efforts would have to be weighed carefully relative to the potential risks of introducing locally maladaptive variation [47].

### Targets of positive directional selection

Connections between broad ecological patterns and the genetic structure of populations are often intuitive and predictable. Upwards range contraction in *T*. *alpinus* is associated with reduced connectivity between suitable montane habitats [33], which we infer has reduced migration between patches and increased genetic drift. However, *a priori* expectations for patterns of recent adaptive evolution are far less predictable in these species. Recent range shifts [20] and temporal changes in diet and skull morphologies [27, 30] are both consistent with stronger directional selection gradients in YNP and SS *T*. *alpinus* relative to *T*. *speciosus*. On the other hand, lower effective population sizes and reduced migration (at least in YNP) should make selection relatively less effective in *T*. *alpinus*. Likewise, more effective adaptive responses could explain why larger and more connected populations of *T*. *speciosus* have remained stable in the face of common environmental stressors.

**Fig 4:**
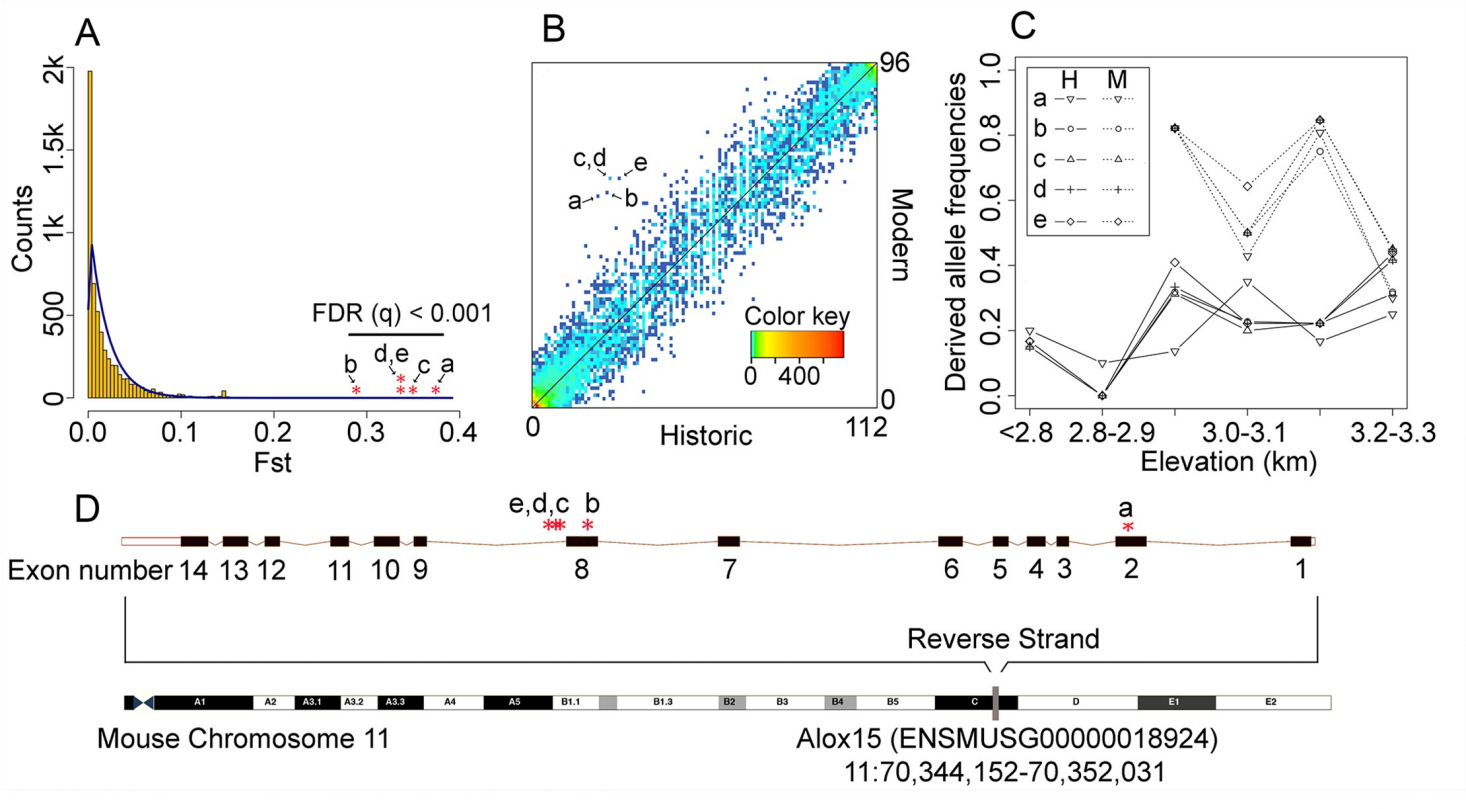
Derived alleles showing significant frequency shifts between historic and modern populations of *Tamias alpinus* in YNP. **(A)** Five outlier SNPs (a-e, FDR q < 0.001) are labeled on a plot of the neutral per site temporal F_ST_ distribution (modern versus historic) estimated by OutFLANK. The histogram of observed F_ST_ (yellow bins) is shown with the inferred neutral distribution (blue line). **(B)** Unfolded two-dimensional site frequency spectrum (2D-SFS) for SNPs between historic (x-axis) and modern (y-axis) YNP *Tamias alpinus* specimens. The color of each data point represents the number of SNPs (depicted by the color key) belonging to that particular 2D-SFS category. Arrows point to the five outliers (a-e) showing the only significant allele frequency shifts over time. **(C)** Derived allele frequencies of the five outliers SNPs plotted against sample elevation. Individual sample localities were pooled into 100-meter elevational bands to enable allele frequency estimation. **(D)** The position of the five outliers mapped onto the *Alox15* gene in the *Mus musculus* reference genome. SNP ‘a’ is a synonymous mutation (A/G) in exon 2 (Chromosome 11:70350801); SNP ‘b’ is synonymous (T/C) and maps to exon 8 (11:70347260); SNPs ‘c’ (T/A), ‘d’ (T/G), and ‘e’ (T/G) are located in the intron between exon 8 and 9.

To begin to tease these issues apart, we tested for specific genetic changes that might underlie recent adaptive responses in these species by directly comparing genetic differences between historic and modern populations. All three temporal population pairs are very closely related (Fig 2), however, they are also separated by changes in population structure and sizes (Fig 3) that may confound standard signatures of positive selection [48]. Therefore, we tested for individual SNPs that had undergone large frequency shifts between historic and modern populations using an approach that is robust to the confounding influence of complex population histories on the genomic distribution of F_ST_ [49–51]. We found no significant allele frequency shifts over time in YNP *T*. *speciosus* or SS *T*. *alpinus*. In contrast, we identified five outlier SNPs in YNP *T*. *alpinus* populations (false discovery rate [FDR] q-value < 0.01) relative to the inferred null distribution of per-site, genome-wide F_ST_ between the temporally sampled populations (genome-wide temporal F_ST_ = 0.012; Fig 4A). To verify the inference of positive selection on these SNPs, we compared the observed F_ST_ values to null distributions simulated under the best ABC-fitted demographic history for YNP *T*. *alpinus* (Fig 3). Our simulated F_ST_ distributions were in close agreement with the overall observed F_ST_ values. Thus, it is very unlikely that demography alone could produce the extreme changes in allele frequencies that we observed at the outlier loci (p-value < 3e-7; S9 Fig).

Derived allele frequencies at all five differentiated SNP positions increased ~threefold in the modern populations (average frequencies of 0.22 historic versus 0.65 modern; Fig 4B) and all were located in the protein-coding gene, Arachidonate 15-Lipoxygenase (*Alox15*) (Fig 4D). *Alox15* is a broadly expressed lipoxygenase involved in the resolution of acute inflammation through the generation of lipid-derived signaling molecules known as resolvins [52–54]. *Alox15* expression has been associated with cardiovascular disease, oxidative stress, and response to hypoxia [55–57] as part of the Hypoxia-inducible factor-1α (HIF-1α) regulation pathway [58]. Two of the outliers represent synonymous changes in non-adjacent exons (positions a, b; Fig 4D) while the three other SNPs (positions c-e) were at non-coding positions within the same intron. All five positions were in strong linkage disequilibrium (historic r^2^ = 0.86; modern r^2^ = 0.93) in YNP *T*. *alpinus* but invariant in all other populations except for one site (b) that was at similar frequency across the SS *T*. *alpinus* temporal contrast (historic = 0.13, modern = 0.2).

Given an ~500m contraction of the low elevation range limit in YNP *T*. *alpinus* over the last century [20], the temporal allele frequency shifts at *Alox15* could simply reflect non-sampling of extinct low elevation populations. To test this, we first estimated *Alox15* allele frequencies as a function of elevation by pooling individuals into discrete 100-meter elevation bands. We observed the largest increases in derived allele frequencies at low to mid-elevation localities (Fig 4C), and mean derived allele frequencies were not significantly correlated with elevation in either of the temporal samples (historical *R*^*2*^ = 0.46, p-value = 0.14; modern *R*^*2*^ = 0.34, p-value = 0.42). Furthermore, all five positions remained strong outliers in our temporal F_ST_ contrasts when we excluded low elevation sampling localities that were present only in the historic YNP *T*. *alpinus* transect (OutFLANK FDR q < 0.05, ABC-fitted F_ST_ distribution p-value = 4e-7). Thus, evolutionary responses at *Alox15* are consistent with *in situ* evolutionary change primarily among remnant demes below the upper bound of the modern YNP *T*. *alpinus* range (<3200 meters elevation).

In principle, the large shift in *Alox15* allele frequencies observed between historic and modern samples could be driven by changes in habitats, food availability, or some other non-climate related environmental factor. However, based on previous modeling of changes in the elevational range [28] and the function of *Alox15*, we suggest that physiological response to warming is the strongest current hypothesis. Winter temperature appears to be a primary limiting factor in the distribution of *T*. *alpinus*, with range contractions strongly tracking upslope shifts in minimum winter temperatures [28]. Increases in minimum winter temperatures at mid-elevations are resulting in reduced YNP snowpacks [59, 60], which Rubidge and colleagues suggested might reduce over-winter survival of *T*. *alpinus* through loss of critical thermal insulation of hibernacula [28]. Interestingly, arousal from hibernation has been shown to induce oxidative stress and hypoxia [61] and *Alox15* shows strong seasonal induction in other species of hibernating squirrels [62].

A potential link between the intensity of selection on variation at *Alox15* and changes in winter snowpack could also explain why we did not detect selection at the same gene in the SS transect. *Tamias alpinus* populations in the Southern Sierra are fixed for ancestral alleles at all but one of the outlier YNP SNPs, suggesting that these populations may lack genetic variation at *Alox15* that is putatively adaptive in YNP. Moreover, SS *T*. *alpinus* populations are currently found above ~3200 meters—above the elevation range showing the largest allele frequency shifts in YNP—and overall snowpack has been more stable in the southern Sierra during the last century [59]. Though speculative, these scenarios help illustrate how evolutionary responses among populations may depend on both adaptive potential (i.e., standing genetic variation) and local environmental conditions.

## Conclusions

Temporal sampling of genomic data has the potential to provide powerful insights into the evolutionary effects of rapid environmental change [18]. Here we built on previous works [1, 9, 33] by generating targeted genome-wide sequence data from 294 chipmunks spanning a century of climate change. By integrating high throughput sequencing, cost and time-effective targeted enrichment technologies, and sophisticated inference methods, we provide powerful comparative insights into demographic and evolutionary responses of two montane species experiencing rapid environmental change. Our genomic time-series approach demonstrates one way that historical archives can be used to study biological responses during recent environmental change [9, 11, 18]. Temporal genomic data can provide a means to understand the current state of populations and their potential evolutionary trajectories, providing powerful tools to inform the conservation of populations experiencing changing environments.

The identification of targets of positive selection during the recent upslope range contraction in *T*. *alpinus* points to a candidate gene and potential phenotypes associated with physiological stress that warrant further study. We caution that further evidence, such as differences in over-winter survival across genotypes or other functional studies, are necessary to demonstrate a causal relationship between *Alox15* and response to climatic-induced stress. Further, our capture experiment only covered a subset of protein coding genes (~50%) and did not include extensive coverage of regulatory regions that may often modulate rapid evolutionary responses [63]. That said, alpine chipmunks also show greater stress response to changes in external conditions [64], a narrower range of activity patterns [31], and more pronounced shifts in diet and functional aspects of cranial morphology when compared to *T*. *speciosus* over the past century [27, 30]. Thus, the combination of phenotypic, behavioral, and now genetic evidence points to some component of physiological stress as a key factor in the greater sensitivity of *T*. *alpinus* to environmental change.

Even in the absence of links to specific phenotypes or fitness, the identification of evolutionary responses at specific genes should help inform future on-ground studies focused on identifying the proximate causes of warming-related population declines across the range of this or other affected species [32]. Indeed, the potential for adaptive evolution to rescue populations in decline has emerged as an important concept in conservation biology [65], with increasing efforts to directly incorporate evolutionary principles into conservation planning [66]. As a cautionary note, our results suggest that putatively adaptive responses in *T*. *alpinus* at *Alox15* (Fig 4), as well as rapid shifts in functional morphology and diet [27, 30], have nonetheless been insufficient to prevent extensive extirpation of lower elevation populations of this alpine specialist.

Comparative analyses of species range shifts over the past century have provided powerful insights into the ecological impacts of and biological responses to rapid environmental changes [1, 19, 21–23]. Here we have begun to extend these ideas to a comparative population genomic framework. Moving forward, we suggest that the true power of analyzing genomes and phenotypes of historical museum archives lies in the potential to extend across species [9, 11]. Though the occurrence of museum records tend to be highly punctuated through space and time for a given species, historic collection efforts, such as those led by Joseph Grinnell and other early naturalists, usually surveyed many co-distributed species. With comparable contemporary sampling efforts, these invaluable archives will enable comparative community level insights into the impacts of and evolutionary responses to rapidly changing environments.

## Methods

### Ethics statement

All animals sampled in the modern era were collected following procedures approved by the University of California, Berkeley Animal Care and Use Committee (Permit number R278–0315). Permits were provided by Yosemite National Park and Sequoia-Kings Canyon National Park.

### Biological samples

*Tamias speciosus* and *T*. *alpinus* surveyed in this study were collected from montane transects in Yosemite National Park (YNP) and the Southern Sierras (SS). Historic samples were collected by Joseph Grinnell and his colleagues from 1911 to 1916, and are preserved as dried skins in the Museum of Vertebrate Zoology (MVZ), at the University of California, Berkeley. Modern samples were collected from the same sites by the ‘Grinnell Resurvey’ team led by MVZ researchers and collaborators from 2003 to 2012 (Fig 1; S1 Table). We examined 100 YNP *T*. *speciosus* (52 historic, 48 modern), 104 YNP *T*. *alpinus* (56 historic, 48 modern), and 90 SS *T*. *alpinus* (52 historic, 38 modern) from each transect. We also sampled six *T*. *minimus* (the Least chipmunk) collected east of YNP, which were used to test for potential hybridization between *T*. *alpinus* and *T*. *minimus* [38]. Furthermore, we included one sample each of three other species (*T*. *striatus*, *T*. *ruficaudus*, and *T*. *amoenus*) in order to polarize SNPs identified in our focal populations. Historic DNA was extracted from toe pad tissue (~3 x 3 mm) in a separate dedicated laboratory using a previously described protocol [9]. DNA was extracted from modern samples using Qiagen DNeasy Blood and Tissue kits following the manufacturer’s protocol. Genomic libraries for all samples were constructed following Meyer and Kircher [67] with slight modifications [9].

### Exome capture design and implementation

We used RNA-seq [68] to sequence and assemble [69] transcriptomes for multiple tissues sampled from a single modern SS *T*. *alpinus* to serve as a reference for exome capture probe design. We targeted exonic regions (6.9 Mb, including flanking introns and intergenic regions) corresponding to 8,053 *T*. *alpinus* genes targeted by our previous array-based capture experiments in chipmunks [9, 70, 71]. In addition, we extracted a broad set of candidate genes from the AmiGO and NCBI protein databases with functional annotations that were potentially relevant to environmental stress responses (e.g., HSP/HSF, hemoglobin, cytokines, apoptosis, immunity, oxidative stress, oxidative phosphorylation). We then used a BLASTx search against these genes to locate 2,054 orthologous transcripts (2.4 Mb) from the *Tamias* transcriptome and included these transcripts in our capture. We also targeted the complete mitochondrial genome (~16.4 Kb) to assess empirical error rates and five previously sequenced nuclear genes [42, 72] to use as positive controls in post-capture qPCR assays of global enrichment efficiency. Probes were designed and manufactured by NimbleGen (SeqCap EZ Developer kits).

Barcoded genomic libraries were pooled together and hybridized in seven independent reactions with *Tamias* Cot-1 DNA and barcode-specific blocking oligonucleotides. Six hybridization experiments were used for the focal species (one per time point for each of the three temporal contrasts) and one additional capture was performed on pooled libraries from six *T*. *minimus* and three outgroup samples (*T*. *striatus*, *T*. *ruficaudus*, and *T*. *amoenus*). After hybridization, each of the enriched genomic libraries were amplified using PCR and sequenced using one lane of Illumina HiSeq2000 per capture (100-bp paired-end).

### Data processing

Bioinformatic processing of exon capture data followed our previous protocols [9, 70]. All raw sequencing reads were treated to remove adapters, exact duplicates, low complexity (i.e., runs of ambiguous or mononucleotide sequence), and reads sourced from bacteria and human contamination. Overlapping paired reads were merged to avoid inflated estimates of coverage and biased genotype likelihoods. We used filtered sequencing reads (28.9 Gb) from 48 modern YNP *T*. *alpinus* samples to generate *de novo* assemblies with ABySS [73] that were then merged using Blat [74], CD-HIT [75], and CAP3 [76] to remove redundancies. This total assembly was then compared to the original targets to construct a non-redundant target reference of 21,128 assembled contiguous sequences (contigs) totaling 20.8 Mb, and error-corrected following Bi and colleagues [9]. We then aligned cleaned reads from *T*. *alpinus*, *T*. *speciosus*, and *T*. *minimus* samples to the *T*. *alpinus* reference using Novoalign (http://www.novocraft.com). Nucleotide positions were filtered at individual, contiguous sequence, and position levels of quality control following our previously described methods [9] (S3 Table) using the script snpCleaner (https://github.com/tplinderoth/ngsQC/tree/master/snpCleaner). For each of the three temporal transects, we retained the intersection of filtered contigs between all historic and modern populations. As a result, 2,569, 2,451, and 2,738 contigs (11.6–13% of the total) were eliminated from YNP *T*. *speciosus*, YNP *T*. *alpinus*, and SS *T*. *alpinus* datasets, respectively. At the site level, we removed sites showing unusually high or low coverage, excessive strand bias, end distance bias, base quality bias, and map quality bias. We also filtered out sites with extensive missing data among samples within each population.

We were particularly attentive to errors associated with long-term DNA degradation. Postmortem nucleotide damage from hydrolytic deamination causes conversion from cytosine (C) to uracil (U) residues resulting in misincorporation of thymine (T) during PCR amplification [34, 35, 77]. We conservatively removed all C-to-T and G-to-A (i.e., the reverse complement of the C-to-T change with respect to the original PCR template molecules) SNP positions from the datasets to avoid inaccurate population genetic inferences stemming from base misincorporation. In total, 9.0, 9.3, and 8.5 Mb of data from YNP *T*. *speciosus*, YNP *T*. *alpinus*, and SS *T*. *alpinus* passed all quality controls and were used in subsequent analyses.

### Population genetic analyses

We used probabilistic methods for variant discovery and allele frequency estimation as implemented within ANGSD [78]. Using a population-specific SFS estimated from allele frequency likelihoods as a prior, we obtained allele frequency posterior probabilities and called SNPs using a 0.95 probability cutoff of being variable. The realSFS function was used to generate 1,000 bootstrap replicates of the folded site frequency spectrum (SFS) for each metapopulation by resampling per site allele frequency likelihoods. We then used ANGSD to estimate the number of segregating sites (S), Watterson's theta (θ_W_), pairwise nucleotide diversity (θπ), and Tajima's *D* in the historic and modern *T*. *alpinus* and *T*. *speciosus* metapopulations. For each metapopulation, we generated 100 estimates of θ_W_ and θπ using randomly chosen SFS bootstrap replicates as priors to evaluate sensitivity of these point estimates on the SFS prior. Additionally we estimated diversity statistics for demes within metapopulations, the former representing spatially clustered sampling localities. For each transect, population differentiation within and between the modern and historic populations was determined using probabilistic methods for estimating F_ST_ [79] and individual covariance matrices for principal component analysis (PCA) as implemented in ngsTools [80]. Confidence intervals (0.95) for global F_ST_ were generated from 1,000 bootstrap replicates of per-site F_ST_ values. To compare allele frequencies over time, we estimated the 2D-SFS between the pooled modern and pooled historic demes of each transect (i.e., three 2D-SFS comparisons). SNPs identified in *T*. *speciosus* and *T*. *alpinus* were polarized relative to *T*. *striatus*, *T*. *ruficaudus*, and *T*. *amoenus*. We further examined population genetic structure using NGSadmix [37], which estimates admixture proportions from genotype likelihoods. We ran 10 replicates for *K* (number of clusters) ranging from 1–10 and summarized results (S5 Table) across runs to determine the best *K* [81]. To test for hybridization between *T*. *alpinus* and *T*. *minimus* samples, we used the program ADMIXTURE [82] to estimate individual ancestries using one randomly sampled SNP per contig.

Next we developed an ABC framework for fitting binned 2D-SFS from serially sampled populations or metapopulations (S4 Fig) and used this approach to test hypotheses about the demographic histories of the sampled chipmunk populations. Additional details on demographic model construction, simulations, model selection, and inference are provided in S1 Text. Briefly, we fitted 5–9 explicit demographic models (S6 Fig) characterized by possible changes in migration and population size to each of the temporal contrasts. We performed 25,000 simulations per model, drawing parameter values from uniform or log-uniform prior distributions and then simulating ~20.2 Mb of sequence data for each individual under the specified history using the coalescent simulator fastsimcoal [83]. Lineages from the different demes were sampled at the present (modern sample) and 90 generations in the past (historic sample) according to the actual number of sampled individuals. Then all samples within a respective time period were pooled and the historic versus modern 2D-SFS was calculated. A custom script was used to calculate diagonal and off diagonal bins of the joint SFS (S4 Fig), which served as our ABC summary statistic. We used the R package 'abc' [84] to calculate model posterior probabilities and to evaluate the reliability of our model selection procedure. We considered the best fitting models for each species/transect to be those with the highest posterior probabilities (S7 Table) and we used a cross validation procedure to determine error rates associated with model choice (S8 Table). To aid model choice, we also considered the fit of the maximum likelihood (ML) estimate for each model to our observed data. We evaluated goodness-of-fit for the selected models by comparing the Euclidean distance between our observed and simulated 2D-SFS bins (S9 Table).

We considered SNPs with large allele frequency shifts between the modern and historic time periods that could not be attributed to demography as evidence for positive selection. We used the program OutFLANK [50] to detect F_ST_ outlier SNPs (FDR q-value < 0.01), empirically adjusting the degrees of freedom of χ^2^-distributed F_ST_ values to account for the influence of demography. We then compared the observed SNP F_ST_ values to null exome-wide and per-site F_ST_ distributions generated by performing 1,500 neutral simulations under the best fitting population history for YNP *T*. *alpinus*.

## Supporting information

S1 Text Approximate Bayesian computation inference. (PDF)

S1 Fig Patterns of base misincorporations in historic samples. The frequencies of the 12 types of substitutions (y-axis) are plotted as a function of distance from the 5′ and 3′-ends of the DNA molecules (x-axis). The first 50 bp of the reads are shown. The substitution frequency of each particular type is calculated as the proportion of a particular alternative (non-reference) base type at a given site along the read, and is coded in different colors and line patterns as indicated at the top of the plots: “X-> Y” indicates a change from reference base type X to alternative base type Y.
(PDF)

S2 Fig Per deme estimates of nucleotide diversity for historical and modern samples of *T*. *speciosus* and *T*. *alpinus*. Estimates represented by a single point reflect demes that were only sampled at a single timepoint.
(PDF)

S3 Fig Genetic ancestry composition of three chipmunk species. Each individual specimen is represented by a vertical bar partitioned into colored segments indicating their proportion of ancestry from each species. Results are shown for modern samples.
(PDF)

S4 Fig Demographic inference procedure. Diagram of the demographic inference method based on fitting bins of the 2D-SFS with Approximate Bayesian Computation (ABC) that was used to infer the *Tamias* population histories. The ABC summary statistics calculated in step 3 are the off-diagonal (left 2D-SFS) and diagonal (right 2D-SFS) bins of the 2D-SFS, where each bin's value is the sum of the counts contained within the bin. The bin width refers to the number of 2D-SFS categories on either side of the diagonal/off-diagonal that are included in the bin and determines the amount of resolution (finer bins for higher resolution) and noise dampening (wider bins) when fitting the 2D-SFS. We used a bin width of 2 in the current study. For assessing model fit in step 5, D_ML,obs_ is the Euclidean distance between the observed or pseudo observed 2D-SFS bins and bins from the maximum likelihood (ML) history under the chosen model. Pseudo observed values are a set of bins under the ML history that are treated like the observed bins.
(PDF)

S5 Fig Unfolded and folded two-dimensional site frequency spectrum (2D-SFS) for SNPs between historic (x-axis) and modern (y-axis) specimens. The color of each data point represents the number of SNPs belonging to that particular category in the 2D-SFS, which is depicted in the color key inset.
(PDF)

S6 Fig Demographic models fit with ABC. The general demographic models that were fit with ABC included histories with demes bottlenecking into the present **(A, B, G)**, historic population expansion **(C, D, J)**, historic population expansion followed by a bottleneck **(H)**, and constant deme size **(E, F, N)**. The shaded circles within the history outlines represent different demes. The actual number of simulated demes equaled the number of sampling localities, which were 10 for YNP *T*. *alpinus*, 3 for SS *T*. *alpinus*, and 8 for YNP *T*. *speciosus*. Demes were simulated under an island model (symmetric migration and identical deme sizes). Per generation migration rates (m) among all deme pairs were equal for the YNP *T*. *alpinus* and *T*. *speciosus* contrasts, while for SS *T*. *alpinus* the migration rates between different pairs of demes were allowed to differ. Solid versus dotted arrows between demes represent potentially different migration rates, while no arrows represent no migration between demes. The parameter m_hist_ is the historic migration rate between two demes, m_mod_ is the modern migration rate between a pair of demes, t_mig_change_ is the number of generations in the past at which the migration rate changes, r_grow_ is the intrinsic growth rate for population expansion, t_grow_stop_ is the number of generations in the past that expansion stops, r_shrink_ is the intrinsic growth rate of population decline, t_shrink_ is the number of generations in the past that a bottleneck starts, and 'mod N_e_' is the effective size of each deme at the present. The presence of ‘90’ in the models involving migration rate change and/or a population bottleneck indicates that these demographic changes were set to occur 90 generations prior to the modern sample.
(PDF)

S7 Fig Assessment of model fit for inferred *Tamias* chipmunk demographic histories. **(A)** Comparison of the empirical cumulative distribution functions (ECDF) for the Euclidean distance between the observed and expected 2D-SFS bins, d(ML,obs), for all YNP *T*. *alpinus* (YNPA), SS *T*. *alpinus* (SSA), and YNP *T*. *speciosus* (YNPS) maximum likelihood histories. For each model, 1,000 simulations under its ML history were performed to generate the expected joint frequency spectra from which the distribution of d(ML,obs) was calculated. Histories with left-shifted ECDF curves are more likely to resemble the true demographic history. **(B-D)** Comparison of D_ML,obs_ and D_ML,pseudo_ distributions for the best-fitting histories for YNP *T*. *alpinus* **(B)**, SS *T*. *alpinus* **(C)**, and YNP *T*. *speciosus* **(D)**. D_ML,obs_ is the Euclidean distance between the observed and expected joint SFS bins under a model's ML history, while D_ML,pseudo_ is the distance between a single set of joint SFS bins under the ML history (pseudo observed) and the expected joint SFS bins. For each ML history, 1,000 simulations were performed to generate the expected joint spectra. The values within the distributions are Weitzman's coefficient of overlapping (OVL), ranging from 0 to 1, which quantifies the area of overlap of the two distributions. More overlap between the two distributions indicates that the ML history is more likely to represent the true demography.
(PDF)

S8 Fig Principal Component Analysis (PCA) plots based on genetic covariance among individuals. The first 3 principal components (PCs) are shown in **(A-C)**. Each point in the PCA plot represents an individual specimen. The proportion of the genetic variance explained by the first 10 PCs is shown in **(D-F)**.
(PDF)

S9 Fig Expected F_ST_ distribution for YNP *T*. *alpinus* under histories inferred with ABC. The expected distribution of neutral F_ST_ per site and exome-wide under the best fitting demographic histories for YNP *T*. *alpinus* inferred with ABC. Each F_ST_ distribution was generated from 1,500 simulations under the maximum likelihood histories for the best fitting demographic models for YNP *T*. *alpinus*, B, F, H, and N. The positions of the five observed F_ST_ outlier SNPs (see Fig 4) are plotted onto the expected per site F_ST_ distributions.
(PDF)

S1 Table Historic and modern sample information. (XLSX)

S2 Table Exon capture data production information. (XLSX)

S3 Table Individual, contig, and site-level quality filters used for genotyping. (PDF)

S4 Table Population genetic summary statistics calculated with ANGSD and ngsTools on quality-filtered data. (PDF)

S5 Table Population genetic structure inferred with ngsAdmix. (XLSX)

S6 Table ABC demographic parameter posterior and maximum likelihood estimates under the best fitting models for each chipmunk temporal contrast. (XLSX)

S7 Table Demographic model posterior probabilities for three *Tamias* temporal contrasts approximated using the rejection method at a tolerance level of 0.8%. (PDF)

S8 Table Demographic model confusion matrix for the three temporal contrasts generated using cross validation with rejection sampling tolerance set at 0.8%. (XLSX)

S9 Table Pairwise comparisons between D_ML,obs_ empirical CDFs for the different models fit to the three temporal contrasts in terms of Kolmogorov-Smirnov 2-Sample test statistics (blue-labeled rows) and their associated p-values (red-labeled rows). (XLSX)
