# Supplementary material for: Temporal genomic contrasts reveal rapid evolutionary responses in an alpine mammal during recent climate change
Source: PLoS Genet. 2019 May 3;15(5):e1008119. doi: 10.1371/journal.pgen.1008119 (PMC6519841; doi:10.1371/journal.pgen.1008119)
Supplement: S1 Text — (PDF) [file pgen.1008119.s001.pdf]

Supporting Information

Temporal genomic contrasts reveal rapid evolutionary responses in an alpine mammal during recent climate change

Ke Bi[1,2,¶], Tyler Linderoth[1,3,¶], Sonal Singhal[1,3], Dan Vanderpool[4], James L. Patton[1,3], Rasmus Nielsen[3], Craig Moritz[1,3,5], Jeffrey M. Good[4,6,*]

[1]Museum of Vertebrate Zoology, 3101 Valley Life Sciences Building, University of California, Berkeley, California 94720, USA

[2]Computational Genomics Resource Laboratory (CGRL), California Institute for Quantitative Biosciences (QB3), University of California, Berkeley, California 94720, USA

[3]Department of Integrative Biology, 3060 Valley Life Sciences Building, University of California, Berkeley, California 94720, USA

[4]Division of Biological Sciences, University of Montana, Missoula, Montana 59812, USA

[5]Research School of Biology and Centre for Biodiversity Analysis, Australian National University, Canberra, ACT 0200, Australia

[6]Wildlife Biology Program, University of Montana, Missoula, MT 59812, USA

[¶]Authors contributed equally to this work

S1 Text. Approximate Bayesian computation inference.

Tyler Linderoth
Email: tl483@cam.ac.uk

ABC Demographic Models. For *Tamias alpinus* and *T. speciosus* from Yosemite National Park (YNP), we modeled each sampling locality as its own deme in an island model with symmetric migration. Although an island model may not closely reflect the actual dispersal behavior of *T. alpinus* or *T. speciosus*, the number of modeled demes precluded sensibly fitting different pairwise migration rates. *Tamias alpinus* populations in the southern Sierras (SS) were modeled in a similar manner, but given fewer sampling localities pairwise migration rates were allowed to vary between different demes.

We fitted four general model topologies (S6 Fig): Bottleneck (A, B, G), historic expansion (C, D, J), historic expansion followed by a bottleneck (H), and constant size models (E, F, N). For some models (A, G, H, J) migration rates were allowed to change (increase or decrease) and in certain cases migration either definitely (B, C, F) or could potentially (D, E) stop altogether. In models A, B, C, and F we fixed population bottlenecks and/or migration rate

changes to begin 90 generations prior to the modern sampling time (i.e., the approximate timing of the historic sampling) given that we knew that there had been changes in genetic structure between the historic and modern samples of *T. alpinus*. To obtain a more precise timeframe for demographic events, we tested these models against those in which the times could vary (D, E, G, H, J). For YNP *T. alpinus* we tested models A, B, C, D, E, F, G, H, N, for *T. speciosus* we tested A, B, D, E, G, H, J, N, and for SS *T. alpinus* we tested models A, G, H, J, N. Different subsets of our total model set were tested for different temporal contrasts because preliminary results showed that fitting some of the nested models was redundant as the more flexible models converged to the simpler models. The same spectrum of possible histories was fitted for each contrast, but with fewer explicit models in some cases. An overview of the entire ABC workflow used to fit histories is outlined in S4 Fig and scripts for carrying it out are available at https://github.com/tplinderoth/ABCutils (doi: 10.5281/zenodo.2591546).

ABC Simulation. We performed 25,000 simulations under each demographic model with a custom script, fastsimcoal_sampler.pl, where each simulation proceeded as follows: 1) draw demographic parameter values from a uniform or log-uniform (if the range exceeded three orders of magnitude) prior distribution; 2) simulate demes under the chosen parameter values using the coalescent simulator fastsimcoal [1]. We simulated 20.18 MB of sequence, which was equal to the reference sequence length, split evenly among 38 unlinked chromosomes. We assumed no recombination, a mouse-based per site mutation rate of $2.2*10^{-9}$, and a transition bias of 0.725 that was empirically determined from the modern chipmunk populations. Individuals were sampled from demes according to the actual sample sizes at the present (modern sample) and 90 generations in the past (historic sample); 3) All samples within a respective time period were pooled and the folded historic versus modern 2D-SFS was calculated. Diagonal and anti-diagonal bins of this joint SFS were then calculated using a bin width of 2. The bin width refers to the number of joint SFS categories on either side of the diagonal that are included in each bin. The joint SFS was binned in this way to reduce noise caused by trying to fit categories with no or few counts, reduce the dimensionality of the summary statistics, and to ensure that we fit the mass correctly everywhere throughout the spectrum. Binning in this manner allows for fitting the shape of the 2D-SFS, which should be a result of demography (barring selection).

ABC Model Selection. Under the rejection-sampling framework when comparing at least two *a priori* equally likely models, the approximate posterior probability of a model is proportional to its proportion of accepted simulations. We used the R package 'abc' [2] *postpr* function to approximate model posterior probabilities with the rejection method set to accept 0.8% of the simulations. Models with the highest posterior probabilities for each temporal contrast were chosen as the best fitting models (S6 Table, S7 Table). In order to evaluate the error associated with choosing models based on their posterior probabilities, we used the cross validation approach implemented in abc to generate a confusion matrix for each contrast (S8 Table).

Briefly, this method works by randomly selecting one simulated 2D-SFS generated during the ABC procedure and treats it as a pseudo-observation. This pseudo-observed 2D-SFS is used to compute the posterior probability of each of the competing models (i.e., the proportion of accepted simulations from rejection sampling) and is assigned to the model with the highest probability. This was conducted 1,000 times for each model to produce confusion matrices. By using this approach on our set of tested models, which we considered to be all plausible demographic scenarios, we were able to quantify the reliability of our ABC procedure to differentiate between them based on the model posterior probabilities.

To aid in model selection, we also compared models based on the goodness-of-fit of their maximum likelihood estimates (ML), which is the set of parameter values that minimized the Euclidean distance between the observed and expected 2D-SFS bins. ML parameter values for each model were used to perform 1,000 additional simulations with fastsimcoal to encapsulate variance due to randomness under the ML histories, and the Euclidean distances between our observed and simulated 2D-SFS bins, $D_{ML,obs}$, were calculated. This produced a distribution of $D_{ML,obs}$ for each of the models, which were compared using Kolmogorov-Smirnov 2-sample tests (KS test) to determine if models were significantly different with respect to their fit to the observed data. Models that were most probable to minimize $D_{ML,obs}$ fit the data best. We found close agreement between the rank order of the models with the highest posterior probabilities and those with the best ML goodness-of-fit for *T. speciosus* and SS *T. alpinus* (S7 Table, S7 Fig). However, agreement between these two criteria was weaker for YNP *T. alpinus.* Consequently, in order to ensure that we evaluated the possibility of all closest-fitting histories to represent the actual history, we also included the model with the best-fitting ML history for YNP *T. alpinus* among its chosen models.

Based on our confusion matrices (S8 Table), we found considerable misclassification between nested models. When considering the fitted histories for the best fitting models in terms of their parameter posterior distributions or ML estimates, they were quite similar. Thus, fitting multiple nested models in essence corroborated one another in that they tended to converge on a similar history, and for this reason they were difficult to differentiate. We were also fitting models to what are likely very recent demographic changes, and so unless they were very strong, signatures of these changes in the joint site frequency spectrum are expected to be weak.

We evaluated goodness-of-fit for the chosen models based on the similarity between the distributions of $D_{ML,obs}$ and $D_{ML,pseudo}$. $D_{ML,pseudo}$ is calculated exactly the same as $D_{ML,obs}$ except that the 2D-SFS bins simulated under the model's ML history are treated as the pseudo-observed data. Similarity between $D_{ML,obs}$ and $D_{ML,pseudo}$ was quantified using Weitzman's overlapping coefficient (OVL), defined as $OVL = \int_{R_n} \min\{f_1(x), f_2(x)\} \mathrm{d}x$. The OVL quantifies the agreement between two probability distributions, $f_1(x)$and $f_2(x)$, and ranges from 0 (the distributions are disjoint) to 1 (the distributions are equal). The OVL can be interpreted as the sum of two error probabilities [3], which translates into the probability of choosing either the ML history as the probability density function (pdf) for $D_{ML,obs}$ or the true history as the pdf for $D_{ML,pseudo}$. This

implies that in our case when the OVL=1 the ML history for a model produces 2D-SFS bins that are the same as the true history, providing strong evidence that it is likely very similar to the actual history. However, it is important to recognize that the degree to which the OVL can be used to determine how similar an inferred history is to the true history also depends on how identifiable the demographic history is from the 2D-SFS bins. It is possible that other demographic scenarios outside of the realm of what was tested could produce large OVL values. The OVLs were calculated from kernel density estimates for $D_{ML,obs}$ and $D_{ML,pseudo}$ using the same bandwidth for all distributions, which was the average of all the respective $D_{ML,obs}$ and $D_{ML,pseudo}$ bandwidths selected using the Sheather-Jones method. All of the ML model selection was performed using R and a custom script, ABCutils.pl.

We note that in order to account for uncertainty in the parameter values, one could simulate with values sampled from the parameter posterior distributions. While such an approach would fully utilize the Bayesian framework, we argue that our approach of simulating from a model's ML estimate to obtain $D_{ML,obs}$ and $D_{ML,pseudo}$ is preferable. Using goodness-of-fit from point estimates to reject models that may not be close to the true population history may actually be more powerful than using posterior predictive p-value approaches which tend to be more conservative [4]. Furthermore, simulations under model ML estimates has traditionally been effective in population genetics for obtaining p-values for observed statistics [5] as was our goal with the F_{ST} outlier validation. Reliable inference under this framework necessitates that the model ML estimate is accurate, and so in our case, ensuring a good fit of the demographic model ML estimate was important.

ABC Parameter Inference. We obtained posterior probability distributions for model parameters using a standard rejection method. Specifically, from the 25,000 simulations performed under each model, we accepted 8% of simulations that had the smallest Euclidean distance between the observed and expected 2D-SFS bins. The parameter posterior probability distributions are the distributions of parameter values from these accepted simulations. Rejection sampling was performed using the ABCutils.pl script.

YNP *T. alpinus* demography. The best fitting models for YNP *T. alpinus* were B, F, H, and N, which mostly converged on a population history characterized by a relatively small, constant population size and increased fragmentation occurring within 90 generations ago. Models B, F, and N had the highest approximate posterior probabilities (S7 Table), while the distribution of the distance between the observed and expected 2D-SFS bins under the ML history for model H suggested that it was also a relatively good fit (S7 Fig). The ML histories under models B, F, H, and N all produced joint spectra similar to the true history as indicated by OVL values greater than 0.81, with model F having the highest value of 0.89 (S7 Fig).

Posterior median parameter estimates for models B, F, and N and the ML estimate for model H (S6 Table) indicate modern deme effective sizes of around 1,350 individuals for YNP *T. alpinus*. A combination of small intrinsic shrink rates and very recent bottleneck times equated

to no population bottlenecks under models B and H. Population expansion was only possible under model H, but with an intrinsic growth rate of 1.1e-8 under the ML history, the population size would effectively remain constant since glaciers began retreating from the YNP region approximately 10K years ago. With the exception of N (constant migration), the best fitting models specify at least a two order of magnitude decrease in migration from historic rates of approximately 7e-5 (based on model B and F posterior median values and model H's ML value). The ML estimate for model H has migration slowing 20 generations prior to the modern sample, while migration entirely stops under models B and F 90 generations before the modern sample. Model N had a 3-7% worse fitting ML history compared to the other best fitting models (S7 Fig), which offers further support for a history involving increased fragmentation as specified under the better fitting ML histories of B, F, and H.

SS *T. alpinus* demography. The best fitting models for *T. alpinus* in the southern Sierras were A, G, and N, which had both the highest approximate posterior probabilities (S7 Table) and ML histories that produced joint frequency spectra most closely resembling the observed 2D-SFS (S7 Fig). The population history inferred from these three models is one with fragmentation potentially recently increasing among demes and a constant population size that is likely around three times larger than YNP *T. alpinus.* There is, however, some evidence for a recent, weak, population bottleneck. Simulations under the ML histories indicate that models A, G, and N are a significantly better fit to the observed data than the other models (all KS test p-values < 2.2e-16) (S9 Table, S7 Fig).

The parameter posterior medians for models A, G, and N (S6 Table) indicate that SS *T. alpinus* has a modern effective deme size of around 4,600 individuals, which has likely remained constant through time. Histories based the posterior medians for models A and G have an essentially negligible bottleneck of at most a few individuals per deme occurring within the past 90 generations. No population size change is also supported by model N and the ML history for model G, which had the highest OVL of 0.93 (S7 Fig). The 95% credible intervals for the intrinsic shrink rates and bottleneck times for models A and G (S6 Table) do not however exclude the possibility for a population bottleneck, but if one did occur, the parameter posterior distributions indicate that it was weak (r < -3e-4) and likely within the past 100 years. The historic migration rates between adjacent demes {1,2} and {2,3} were around 3e-4 and 6e-5 respectively, while the migration rate was lower between the geographically most distant demes, {1,3}, ranging from possibly around 2e-9 (based on model G and N ML estimates) to 3.5e-7 (based on posterior median estimates). The posterior median values for models A and G indicate that somewhere between 29-90 generations before the modern sample migration rates between demes decreased to around 2e-5, 1e-5, and 2e-8 for demes {1,2}, {2,3}, and {1,3} respectively. While the ML history for model A supports this increased fragmentation among all demes, the ML history for model G has migration changing only 5 generations prior to the modern sample suggesting a history with effectively no change in migration, which is supported by model N.

Taken together, this makes it uncertain whether population fragmentation has changed in the southern Sierras, but if it has, it was likely a subtle increase within the past 100 years.

YNP *T. speciosus* demography. Models D, H, and J had the best fit for YNP *T. speciosus* in terms of both their approximate posterior probabilities (S7 Table) and distance of their ML histories from the observed data (S7 Fig). The fits for these models indicate that *T. specious* is characterized by a past population expansion and a modern effective size that is likely at least three times larger than *T. alpinus* in YNP. The model fits also provide some evidence to suggest that migration among *T. speciosus* demes has also decreased recently. Simulations under the ML histories showed a clear and significant difference (all KS test p-values < 2.2e-16) in the fits between models D, H, and J and the other tested models (S9 Table, S7 Fig).

Based on the parameter posterior medians for models D, H, and J (S6 Table), *T. speciosus* demes were expanding at an intrinsic rate of around 8e-6 until 45 - 1,234 generations ago, at which point deme sizes have remained constant at around 4,560 individuals. Models D and J did not involve a bottleneck, and although model H allowed for one, small shrink rates and very recent bottleneck times equated to no bottleneck based on posterior median and ML parameter estimates. The migration rate posterior medians for models D, H, and J decreased from around 3e-4 to rates of 0 – 1.47e-5 within 33 generations prior to the modern sample. It should be noted that the ML estimates for models D and H indicate that migration rates remain constant at around 7.7e-4, however, the ML estimate under model J, which nearly perfectly fit the true history according to an OVL value of 0.98 (S7 Fig), had migration decreasing from 3.42e-3 to 1.17e-8 341 generations ago. Parameter posterior distributions for all three best fitting models suggest that if migration has decreased, it was likely within the last 100 generations (S6 Table). It is difficult to discern for certain whether fragmentation has increased for YNP *T. speciosus,* but the single, best-fitting, ML history and the posterior median migration rate estimates across all three closest fitting models suggest that it has.

Supporting References

1. Excoffier L, Foll M. fastsimcoal: a continuous-time coalescent simulator of genomic diversity under arbitrarily complex evolutionary scenarios. Bioinformatics. 2011;27:1332-4.
2. Csilléry K, Francois O, Blum MGB. abc: an R package for approximate Bayesian computation (ABC). Methods in Ecology and Evolution. 2012;3:475-9.
3. Schmid F, Schmidt A. Nonparametric estimation of the coefficient of overlapping – theory and empirical application. Computational Statistics & Data Analysis. 2006;50:1583-96.
4. Gelman A. Two simple examples for understanding posterior p-values whose distributions are far from uniform. Electronic Journal of Statistics. 2013;7:2595-602.
5. Goldman N. Statistical tests of models of DNA substitution. Journal of Molecular Evolution. 1993;36:182-98.
