# Supplementary material for: Temporal genomic contrasts reveal rapid evolutionary responses in an alpine mammal during recent climate change
Source: PLoS Genet. 2019 May 3;15(5):e1008119. doi: 10.1371/journal.pgen.1008119 (PMC6519841; doi:10.1371/journal.pgen.1008119)
Supplement: S1 Fig — The frequencies of the 12 types of substitutions (y-axis) are plotted as a function of distance from the 5′ and 3′-ends of the DNA molecules (x-axis). The first 50 bp of the reads are shown. The substitution frequency of each particular type is calculated as the proportion of a particular alternative (non-reference) base type at a given site along the read, and is coded in different colors and line patterns as indicated at the top of the plots: “X-> Y” indicates a change from reference base type X to alternative base type Y. (PDF) [file pgen.1008119.s002.pdf]

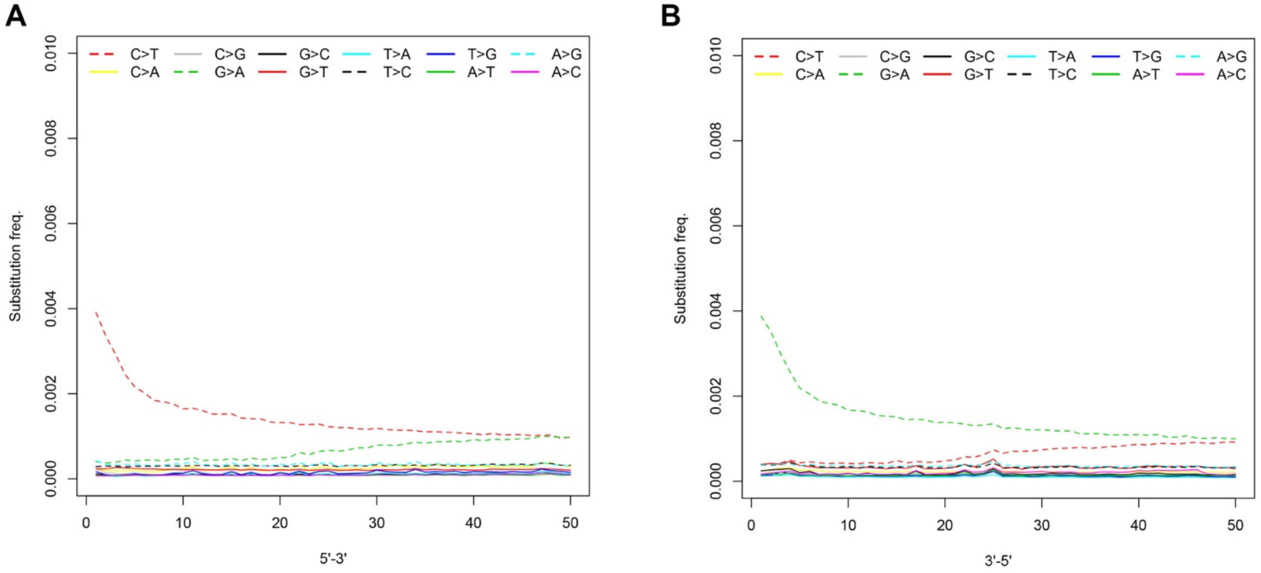

S1 Fig. Patterns of base misincorporations in historic samples. The frequencies of the 12 types of substitutions (y-axis) are plotted as a function of distance from the 5′ and 3′-ends of the DNA molecules (x-axis). The first 50 bp of the reads are shown. The substitution frequency of each particular type is calculated as the proportion of a particular alternative (non-reference) base type at a given site along the read, and is coded in different colors and line patterns as indicated at the top of the plots: “X-> Y” indicates a change from reference base type X to alternative base type Y.
