# Supplementary material for: Temporal genomic contrasts reveal rapid evolutionary responses in an alpine mammal during recent climate change
Source: PLoS Genet. 2019 May 3;15(5):e1008119. doi: 10.1371/journal.pgen.1008119 (PMC6519841; doi:10.1371/journal.pgen.1008119)
Supplement: S2 Fig — Estimates represented by a single point reflect demes that were only sampled at a single timepoint. (PDF) [file pgen.1008119.s003.pdf]

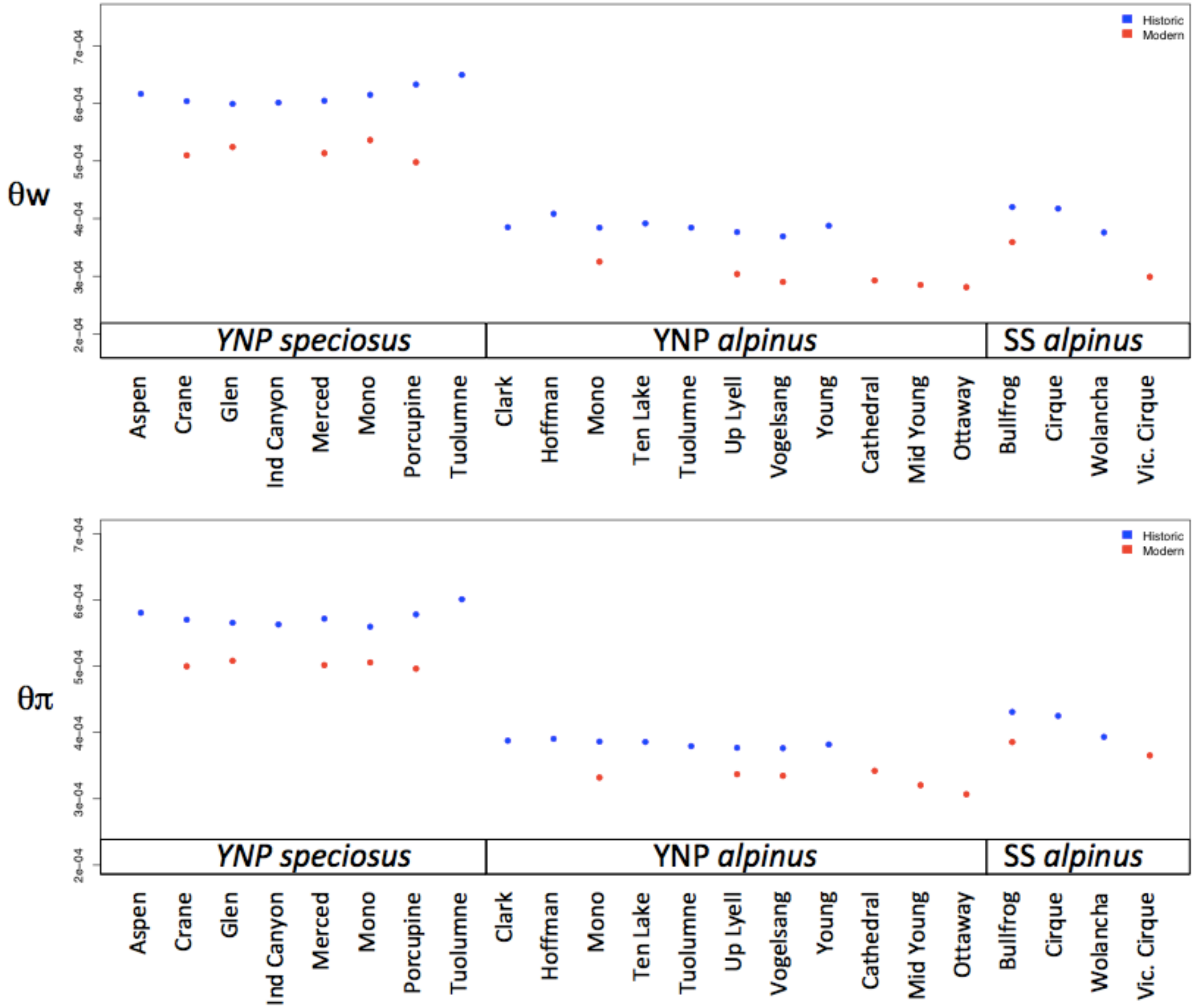

S2 Fig. Per deme estimates of nucleotide diversity for historical and modern samples of *T. speciosus* and *T. alpinus*. Estimates represented by a single point reflect demes that were only sampled at a single timepoint.
