# Supplementary material for: Temporal genomic contrasts reveal rapid evolutionary responses in an alpine mammal during recent climate change
Source: PLoS Genet. 2019 May 3;15(5):e1008119. doi: 10.1371/journal.pgen.1008119 (PMC6519841; doi:10.1371/journal.pgen.1008119)
Supplement: S3 Fig — Each individual specimen is represented by a vertical bar partitioned into colored segments indicating their proportion of ancestry from each species. Results are shown for modern samples. (PDF) [file pgen.1008119.s004.pdf]

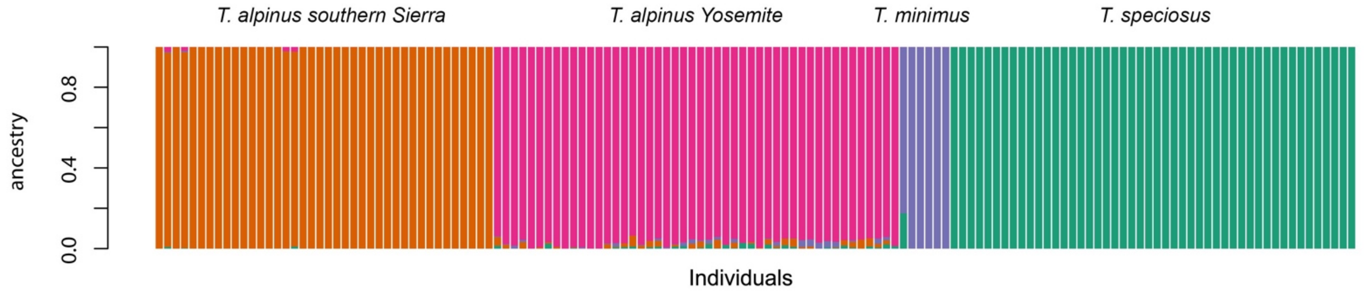

S3 Fig. Genetic ancestry composition of three chipmunk species. Each individual specimen is represented by a vertical bar partitioned into colored segments indicating their proportion of ancestry from each species. Results are shown for modern samples.
