# Supplementary material for: Temporal genomic contrasts reveal rapid evolutionary responses in an alpine mammal during recent climate change
Source: PLoS Genet. 2019 May 3;15(5):e1008119. doi: 10.1371/journal.pgen.1008119 (PMC6519841; doi:10.1371/journal.pgen.1008119)
Supplement: S4 Fig — Diagram of the demographic inference method based on fitting bins of the 2D-SFS with Approximate Bayesian Computation (ABC) that was used to infer the Tamias population histories. The ABC summary statistics calculated in step 3 are the off-diagonal (left 2D-SFS) and diagonal (right 2D-SFS) bins of the 2D-SFS, where each bin's value is the sum of the counts contained within the bin. The bin width refers to the number of 2D-SFS categories on either side of the diagonal/off-diagonal that are included in the bin and determines the amount of resolution (finer bins for higher resolution) and noise dampening (wider bins) when fitting the 2D-SFS. We used a bin width of 2 in the current study. For assessing model fit in step 5, DML,obs is the Euclidean distance between the observed or pseudo observed 2D-SFS bins and bins from the maximum likelihood (ML) history under the chosen model. Pseudo observed values are a set of bins under the ML history that are treated like the observed bins. (PDF) [file pgen.1008119.s005.pdf]

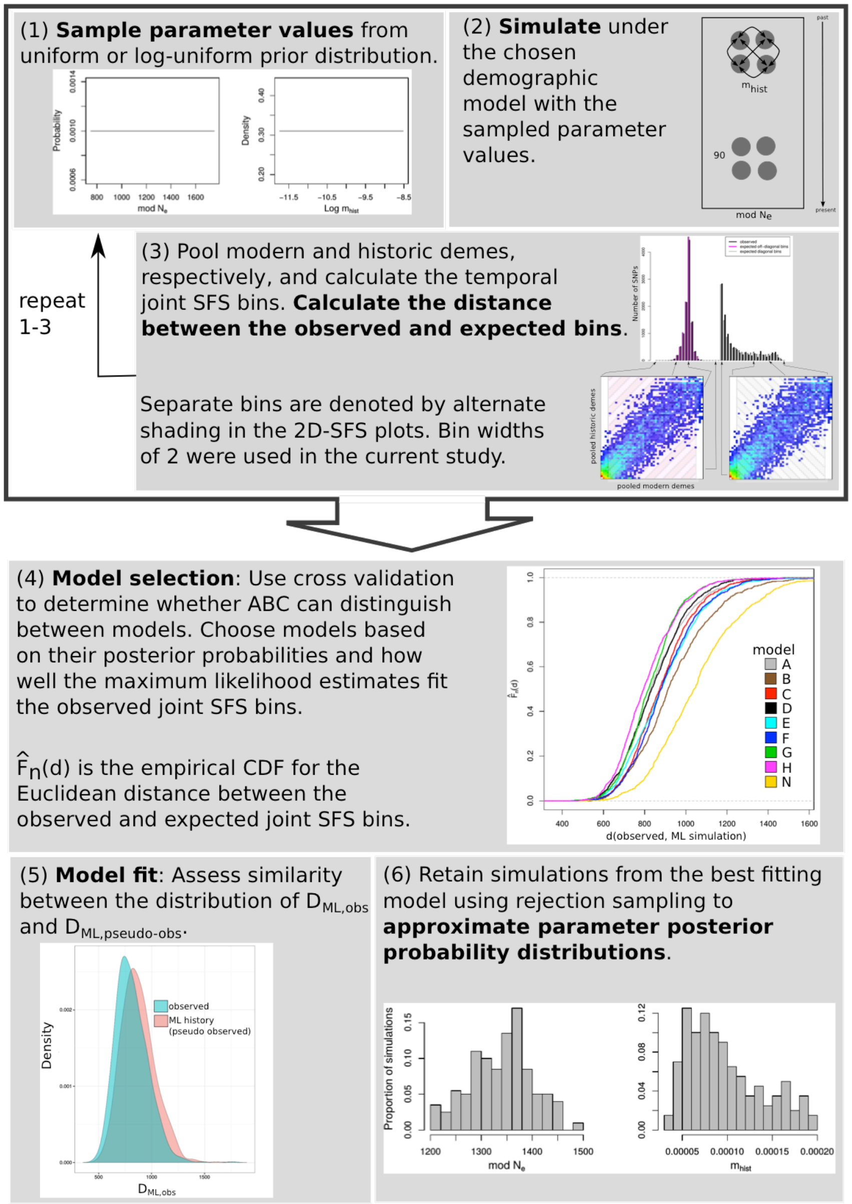

S4 Fig. Demographic inference procedure. Diagram of the demographic inference method based on fitting bins of the 2D-SFS with Approximate Bayesian Computation (ABC) that was used to infer the *Tamias* population histories. The ABC summary statistics calculated in step 3 are the off-diagonal (left 2D-SFS) and diagonal (right 2D-SFS) bins of the 2D-SFS, where each bin's value is the sum of the counts contained within the bin. The bin width refers to the number of 2D-SFS categories on either side of the diagonal/off-diagonal that are included in the bin and determines the amount of resolution (finer bins for higher resolution) and noise dampening (wider bins) when fitting the 2D-SFS. We used a bin width of 2 in the current study. For assessing model fit in step 5, $D_{ML,obs}$ is the Euclidean distance between the observed or pseudo observed 2D-SFS bins and bins from the maximum likelihood (ML) history under the chosen model. Pseudo observed values are a set of bins under the ML history that are treated like the observed bins.
