# Supplementary material for: Temporal genomic contrasts reveal rapid evolutionary responses in an alpine mammal during recent climate change
Source: PLoS Genet. 2019 May 3;15(5):e1008119. doi: 10.1371/journal.pgen.1008119 (PMC6519841; doi:10.1371/journal.pgen.1008119)
Supplement: S5 Fig — The color of each data point represents the number of SNPs belonging to that particular category in the 2D-SFS, which is depicted in the color key inset. (PDF) [file pgen.1008119.s006.pdf]

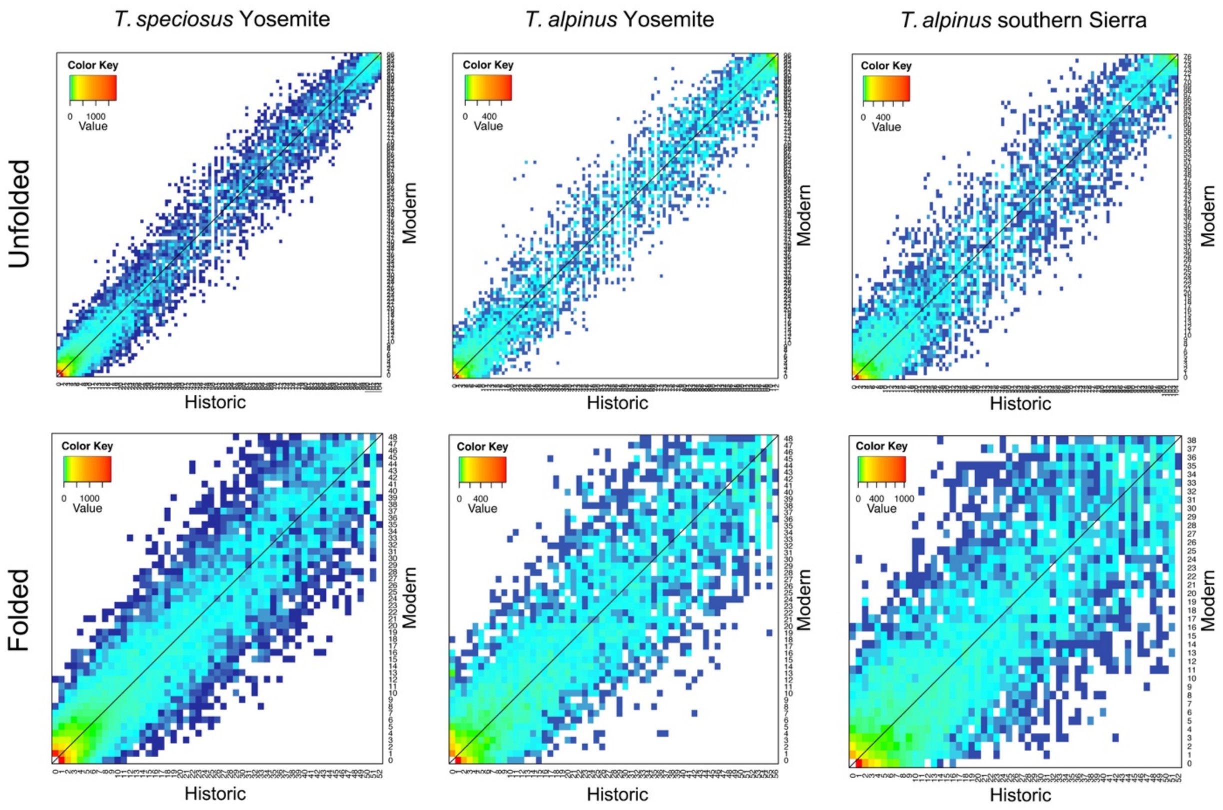

S5 Fig. Unfolded and folded two-dimensional site frequency spectrum (2D-SFS) for SNPs between historic (x-axis) and modern (y-axis) specimens. The color of each data point represents the number of SNPs belonging to that particular category in the 2D-SFS, which is depicted in the color key inset.
