# Supplementary material for: Temporal genomic contrasts reveal rapid evolutionary responses in an alpine mammal during recent climate change
Source: PLoS Genet. 2019 May 3;15(5):e1008119. doi: 10.1371/journal.pgen.1008119 (PMC6519841; doi:10.1371/journal.pgen.1008119)
Supplement: S6 Fig — The general demographic models that were fit with ABC included histories with demes bottlenecking into the present (A, B, G), historic population expansion (C, D, J), historic population expansion followed by a bottleneck (H), and constant deme size (E, F, N). The shaded circles within the history outlines represent different demes. The actual number of simulated demes equaled the number of sampling localities, which were 10 for YNP T. alpinus, 3 for SS T. alpinus, and 8 for YNP T. speciosus. Demes were simulated under an island model (symmetric migration and identical deme sizes). Per generation migration rates (m) among all deme pairs were equal for the YNP T. alpinus and T. speciosus contrasts, while for SS T. alpinus the migration rates between different pairs of demes were allowed to differ. Solid versus dotted arrows between demes represent potentially different migration rates, while no arrows represent no migration between demes. The parameter mhist is the historic migration rate between two demes, mmod is the modern migration rate between a pair of demes, tmig_change is the number of generations in the past at which the migration rate changes, rgrow is the intrinsic growth rate for population expansion, tgrow_stop is the number of generations in the past that expansion stops, rshrink is the intrinsic growth rate of population decline, tshrink is the number of generations in the past that a bottleneck starts, and 'mod Ne' is the effective size of each deme at the present. The presence of ‘90’ in the models involving migration rate change and/or a population bottleneck indicates that these demographic changes were set to occur 90 generations prior to the modern sample. (PDF) [file pgen.1008119.s007.pdf]

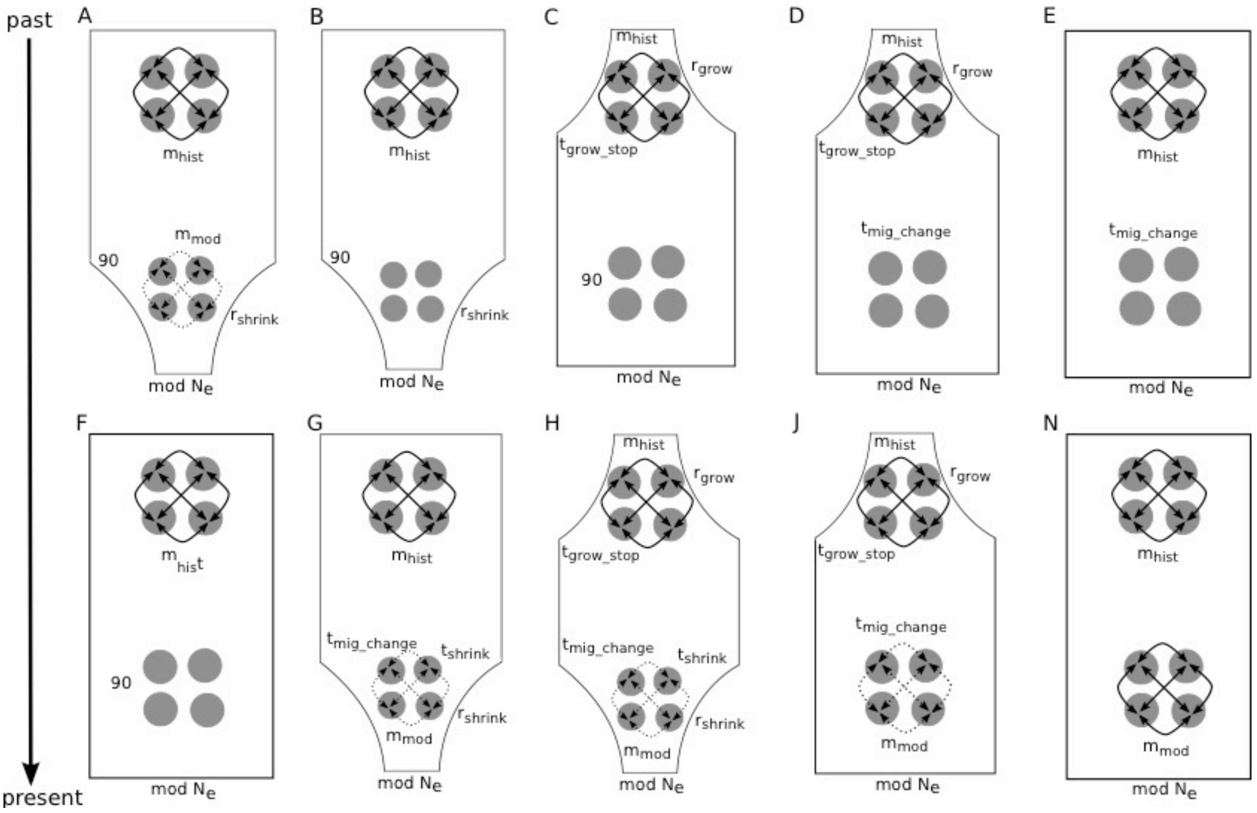


**S6 Fig. Demographic models fit with ABC.** The general demographic models that were fit with ABC included histories with demes bottlenecking into the present **(A, B, G)**, historic population expansion **(C, D, J)**, historic population expansion followed by a bottleneck **(H)**, and constant deme size **(E, F, N)**. The shaded circles within the history outlines represent different demes. The actual number of simulated demes equaled the number of sampling localities, which were 10 for YNP *T. alpinus*, 3 for SS *T. alpinus*, and 8 for YNP *T. speciosus*. Demes were simulated under an island model (symmetric migration and identical deme sizes). Per generation migration rates (m) among all deme pairs were equal for the YNP *T. alpinus* and *T. speciosus* contrasts, while for SS *T. alpinus* the migration rates between different pairs of demes were allowed to differ. Solid versus dotted arrows between demes represent potentially different migration rates, while no arrows represent no migration between demes. The parameter $m_{hist}$ is the historic migration rate between two demes, $m_{mod}$ is the modern migration rate between a pair of demes, $t_{mig\_change}$ is the number of generations in the past at which the migration rate changes, $r_{grow}$ is the intrinsic growth rate for population expansion, $t_{grow\_stop}$ is the number of generations in the past that expansion stops, $r_{shrink}$ is the intrinsic growth rate of population decline, $t_{shrink}$ is the number of generations in the past that a bottleneck starts, and 'mod $N_e$' is the effective size of each deme at the present. The presence of ‘90’ in the models involving migration rate change and/or a population bottleneck indicates that these demographic changes were set to occur 90 generations prior to the modern sample.
