# Supplementary material for: Temporal genomic contrasts reveal rapid evolutionary responses in an alpine mammal during recent climate change
Source: PLoS Genet. 2019 May 3;15(5):e1008119. doi: 10.1371/journal.pgen.1008119 (PMC6519841; doi:10.1371/journal.pgen.1008119)
Supplement: S7 Fig — (A) Comparison of the empirical cumulative distribution functions (ECDF) for the Euclidean distance between the observed and expected 2D-SFS bins, d(ML,obs), for all YNP T. alpinus (YNPA), SS T. alpinus (SSA), and YNP T. speciosus (YNPS) maximum likelihood histories. For each model, 1,000 simulations under its ML history were performed to generate the expected joint frequency spectra from which the distribution of d(ML,obs) was calculated. Histories with left-shifted ECDF curves are more likely to resemble the true demographic history. (B-D) Comparison of DML,obs and DML,pseudo distributions for the best-fitting histories for YNP T. alpinus (B), SS T. alpinus (C), and YNP T. speciosus (D). DML,obs is the Euclidean distance between the observed and expected joint SFS bins under a model's ML history, while DML,pseudo is the distance between a single set of joint SFS bins under the ML history (pseudo observed) and the expected joint SFS bins. For each ML history, 1,000 simulations were performed to generate the expected joint spectra. The values within the distributions are Weitzman's coefficient of overlapping (OVL), ranging from 0 to 1, which quantifies the area of overlap of the two distributions. More overlap between the two distributions indicates that the ML history is more likely to represent the true demography. (PDF) [file pgen.1008119.s008.pdf]

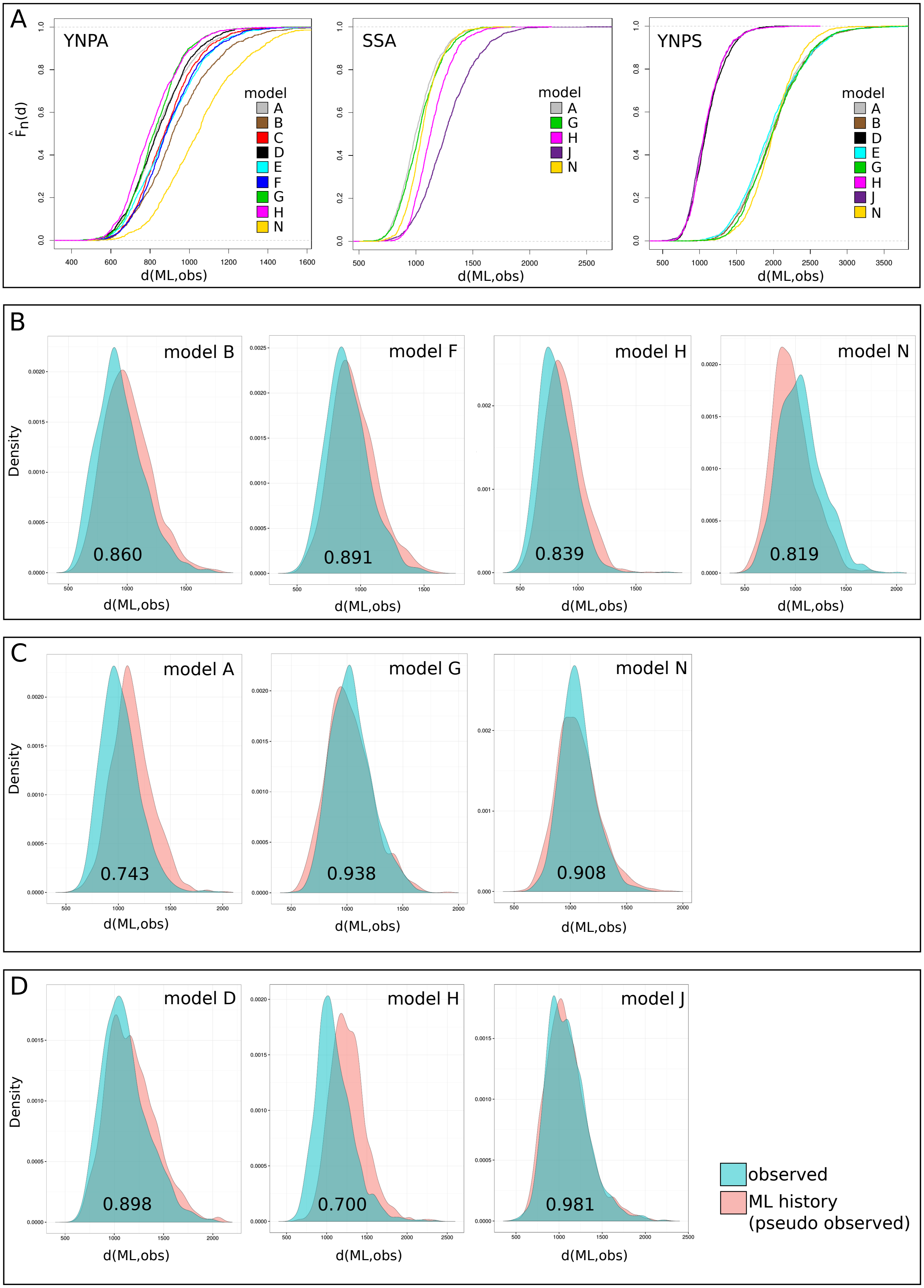
A
YNPA
SSA
YNPS
model
A
B
C
D
E
F
G
H
J
N
d(ML,obs)
B
model B
model F
model H
model N
0.860
0.891
0.839
0.819
Density
C
model A
model G
model N
0.743
0.938
0.908
D
model D
model H
model J
0.898
0.700
0.981
observed
ML history
(pseudo observed)

S7 Fig. Assessment of model fit for inferred *Tamias* chipmunk demographic histories. (A) Comparison of the empirical cumulative distribution functions (ECDF) for the Euclidean distance between the observed and expected 2D-SFS bins, d(ML,obs), for all YNP *T. alpinus* (YNPA), SS *T. alpinus* (SSA), and YNP *T. speciosus* (YNPS) maximum likelihood histories. For each model, 1,000 simulations under its ML history were performed to generate the expected joint frequency spectra from which the distribution of d(ML,obs) was calculated. Histories with left-shifted ECDF curves are more likely to resemble the true demographic history. **(B-D)** Comparison of $D_{ML,obs}$ and $D_{ML,pseudo}$ distributions for the best-fitting histories for YNP *T. alpinus* **(B)**, SS *T. alpinus* **(C)**, and YNP *T. speciosus* **(D)**. $D_{ML,obs}$ is the Euclidean distance between the observed and expected joint SFS bins under a model's ML history, while $D_{ML,pseudo}$ is the distance between a single set of joint SFS bins under the ML history (pseudo observed) and the expected joint SFS bins. For each ML history, 1,000 simulations were performed to generate the expected joint spectra. The values within the distributions are Weitzman's coefficient of overlapping (OVL), ranging from 0 to 1, which quantifies the area of overlap of the two distributions. More overlap between the two distributions indicates that the ML history is more likely to represent the true demography.
