# Supplementary material for: Temporal genomic contrasts reveal rapid evolutionary responses in an alpine mammal during recent climate change
Source: PLoS Genet. 2019 May 3;15(5):e1008119. doi: 10.1371/journal.pgen.1008119 (PMC6519841; doi:10.1371/journal.pgen.1008119)
Supplement: S8 Fig — The first 3 principal components (PCs) are shown in (A-C). Each point in the PCA plot represents an individual specimen. The proportion of the genetic variance explained by the first 10 PCs is shown in (D-F). (PDF) [file pgen.1008119.s009.pdf]

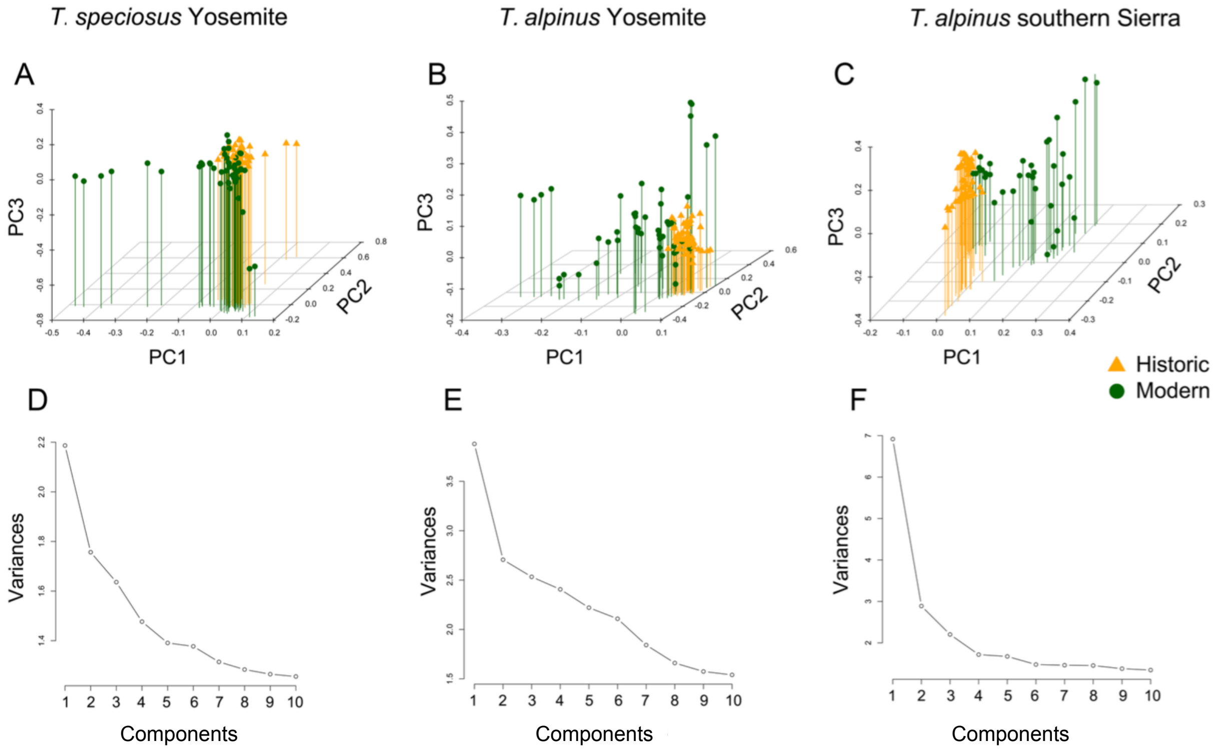

S8 Fig. Principal Component Analysis (PCA) plots based on genetic covariance among individuals. The first 3 principal components (PCs) are shown in **(A-C)**. Each point in the PCA plot represents an individual specimen. The proportion of the genetic variance explained by the first 10 PCs is shown in **(D-F)**.
