# Supplementary material for: Temporal genomic contrasts reveal rapid evolutionary responses in an alpine mammal during recent climate change
Source: PLoS Genet. 2019 May 3;15(5):e1008119. doi: 10.1371/journal.pgen.1008119 (PMC6519841; doi:10.1371/journal.pgen.1008119)
Supplement: S9 Fig — The expected distribution of neutral FST per site and exome-wide under the best fitting demographic histories for YNP T. alpinus inferred with ABC. Each FST distribution was generated from 1,500 simulations under the maximum likelihood histories for the best fitting demographic models for YNP T. alpinus, B, F, H, and N. The positions of the five observed FST outlier SNPs (see Fig 4) are plotted onto the expected per site FST distributions. (PDF) [file pgen.1008119.s010.pdf]

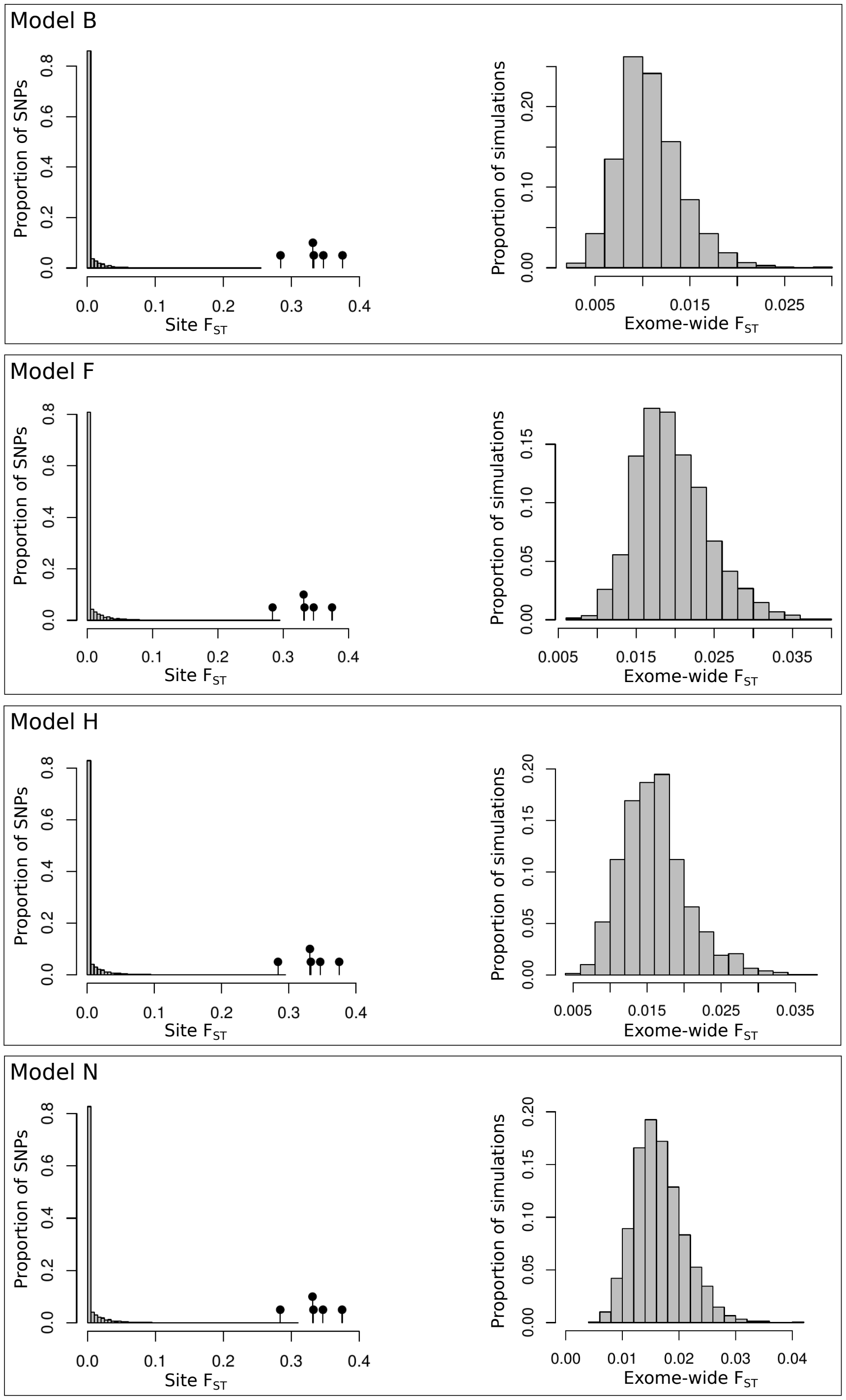

S9 Fig. Expected F_{ST} distribution for YNP *T. alpinus* under histories inferred with ABC. The expected distribution of neutral F_{ST} per site and exome-wide under the best fitting demographic histories for YNP *T. alpinus* inferred with ABC. Each F_{ST} distribution was generated from 1,500 simulations under the maximum likelihood histories for the best fitting demographic models for YNP *T. alpinus*, B, F, H, and N. The positions of the five observed F_{ST} outlier SNPs (see Fig 4) are plotted onto the expected per site F_{ST} distributions.
