# Supplementary material for: Temporal genomic contrasts reveal rapid evolutionary responses in an alpine mammal during recent climate change
Source: PLoS Genet. 2019 May 3;15(5):e1008119. doi: 10.1371/journal.pgen.1008119 (PMC6519841; doi:10.1371/journal.pgen.1008119)
Supplement: S3 Table — (PDF) [file pgen.1008119.s013.pdf]

S3 Table. Individual, contig, and site-level quality filters used for genotyping.

1. Filtering at individual level
(a) Remove individuals having extremely low or high coverage (<1/5 or >5 x the average coverage across all individuals).
(b) Remove individuals with excessively high sequencing error rates measured as the percentage of mismatched bases out of the total number of aligned bases in the mitochondrial genome. (>3X increase in error rates relative to the per individual average – note, no individuals in the current study exceeded this filter threshold)
2. Filtering at contig level
(a) Remove contigs having extremely low (<1st percentile) or high (>99th percentile) coverage based on the empirical coverage distribution across contigs.
(b) Remove contigs with at least one SNP having allele frequencies highly deviating from Hardy–Weinberg equilibrium expectations ($p < 0.0001$) based on a two-tailed exact test.
(c) Retain only the contigs that pass all filters for both historic and contemporary samples
3. Filtering at site level
(a) Remove sites with excessively low (<1st percentile) or high (>99th percentile) coverage based on the empirical coverage distribution.
(b) Remove sites with biases associated with reference and alternative allele Phred quality (1e-100), mapping quality (0), and distance of alleles from the ends of reads (0.0001). Also remove sites that show a bias towards sequencing reads coming from the forward or reverse strand (0.0001).
(c) Remove sites for which there are not at least 80% of the individuals sequenced at a minimum of 3x coverage each.
(d) Remove sites with a Phred scaled root mean square mapping quality for SNPs below 10.
(e) Due to the high base misincorporation rate present in the historic samples, we remove sites from all individuals for which C to T and G to A SNPs are identified.
(f) Retain only the sites that pass all filters for both historic and contemporary samples.
