# Supplementary material for: Temporal genomic contrasts reveal rapid evolutionary responses in an alpine mammal during recent climate change
Source: PLoS Genet. 2019 May 3;15(5):e1008119. doi: 10.1371/journal.pgen.1008119 (PMC6519841; doi:10.1371/journal.pgen.1008119)
Supplement: S4 Table — (PDF) [file pgen.1008119.s014.pdf]

S4 Table. Population genetic summary statistics calculated with ANGSD and ngsTools on quality-filtered data.

Populations	n	S	Private SNP	% θ_W Global	% θ_W Deme	% $\theta\pi$ Global	% $\theta\pi$ Deme	Tajima's *D*	Global F_{ST} (0.95 CI)
YNP *T. speciosus* Historic	52	18309	2977	0.078	0.062	0.057	0.057	-0.342	0.0274 (0.0274-0.0274)
YNP *T. speciosus* Modern	48	17959	2627	0.060	0.052	0.051	0.050	-0.362	0.0296 (0.0296-0.0296)
YNP *T. alpinus* Historic	56	9495	1397	0.042	0.039	0.038	0.038	0.433	0.0324 (0.0324-0.0325)
YNP *T. alpinus* Modern	48	8998	900	0.032	0.030	0.035	0.033	0.518	0.0575 (0.0575-0.0575)
SS *T. alpinus* Historic	52	10211	2232	0.044	0.040	0.043	0.042	0.336	0.0344 (0.0344-0.0345)
SS *T. alpinus* Modern	38	8722	743	0.033	0.033	0.038	0.038	0.516	0.0438 (0.0438-0.0439)

- S = the number of segregating sites
- Private SNP = number of SNP detected only in this population
- θ_W = Watterson's theta, expressed as a percent for the overall metapopulation (Global) and the average value per deme (Deme).
- $\theta\pi$ = Pairwise nucleotide diversity, expressed as a percent for the overall metapopulation (Global) and the average value per deme (Deme).
- Tajima's *D* estimated from the overall metapopulation.
