# Supplementary material for: Temporal genomic contrasts reveal rapid evolutionary responses in an alpine mammal during recent climate change
Source: PLoS Genet. 2019 May 3;15(5):e1008119. doi: 10.1371/journal.pgen.1008119 (PMC6519841; doi:10.1371/journal.pgen.1008119)
Supplement: S7 Table — (PDF) [file pgen.1008119.s017.pdf]

S7 Table. Demographic model posterior probabilities for three *Tamias* temporal contrasts approximated using the rejection method at a tolerance level of 0.8%.

Populations	A	B	C	D	E	F	G	H	J	N
YNP *T. speciosus*	0.0425	0.0231	-	0.2106	0.0619	-	0.0862	0.2394	0.2394	0.0969
YNP *T. alpinus*	0.0311	0.2011	0.0861	0.0667	0.1222	0.1794	0.0561	0.0478	-	0.2094
SS *T. alpinus*	0.2040	-	-	-	-	-	0.2120	0.1780	0.1700	0.2360
